# Viral FGARAT ORF75A promotes early events in lytic infection and gammaherpesvirus pathogenesis in mice

**DOI:** 10.1371/journal.ppat.1006843

**Published:** 2018-02-01

**Authors:** Nick D. Van Skike, Nana K. Minkah, Chad H. Hogan, Gary Wu, Peter T. Benziger, Darby G. Oldenburg, Mehmet Kara, Deborah M. Kim-Holzapfel, Douglas W. White, Scott A. Tibbetts, Jarrod B. French, Laurie T. Krug

**Affiliations:** 1 Department of Molecular Genetics and Microbiology, Stony Brook University, Stony Brook, New York, United States of America; 2 Graduate Program of Genetics, Stony Brook University, Stony Brook, New York, United States of America; 3 Gundersen Health System, La Crosse, Wisconsin, United States of America; 4 Department of Molecular Genetics and Microbiology and UF Shands Cancer Center, College of Medicine, University of Florida, Gainesville, Florida, United States of America; 5 Departments of Chemistry and of Biochemistry and Cell Biology, Stony Brook University, Stony Brook, New York, United States of America; Baylor College of Medicine, UNITED STATES

## Abstract

Gammaherpesviruses encode proteins with homology to the cellular purine metabolic enzyme formyl-glycinamide-phosphoribosyl-amidotransferase (FGARAT), but the role of these viral FGARATs (vFGARATs) in the pathogenesis of a natural host has not been investigated. We report a novel role for the ORF75A vFGARAT of murine gammaherpesvirus 68 (MHV68) in infectious virion production and colonization of mice. MHV68 mutants with premature stop codons in *orf75A* exhibited a log reduction in acute replication in the lungs after intranasal infection, which preceded a defect in colonization of multiple host reservoirs including the mediastinal lymph nodes, peripheral blood mononuclear cells, and the spleen. Intraperitoneal infection rescued splenic latency, but not reactivation. The 75A.stop virus also exhibited defective replication in primary fibroblast and macrophage cells. Viruses produced in the absence of ORF75A were characterized by an increase in the ratio of particles to PFU. In the next round of infection this led to the alteration of early events in lytic replication including the deposition of the ORF75C tegument protein, the accelerated kinetics of viral gene expression, and induction of TNFα release and cell death. Infecting cells to deliver equivalent genomes revealed that ORF75A was required for initiating early events in infection. In contrast with the numerous phenotypes observed in the absence of ORF75A, ORF75B was dispensable for replication and pathogenesis. These studies reveal that murine rhadinovirus vFGARAT family members ORF75A and ORF75C have evolved to perform divergent functions that promote replication and colonization of the host.

## Author summary

Gammaherpesviruses are infectious agents that cause cancer. The study of viral genes unique to this subfamily may offer insight into the strategies that these viruses use to persist in the host and drive disease. The vFGARATs are a family of viral proteins found only in gammaherpesviruses, and are critical for replication in cell culture. Here we report that a rhadinovirus of rodents requires a previously uncharacterized vFGARAT family member, ORF75A, to support viral growth and persistence in mice. In addition, viruses lacking ORF75A are defective in the production of infectious viral particles. Thus, duplications and functional divergence of the various vFGARATs in the rhadinovirus lineage have likely been driven by selective pressures to disseminate within and colonize the host. Identification of the shared host processes that are targeted by the diverse family of vFGARATs may reveal novel targets for therapeutic agents to prevent life-long infections by these oncogenic viruses.

## Introduction

Herpesviruses traverse multiple cell types to ultimately gain access to host cells that serve as long-term reservoirs of latent infection. The successful colonization and maintenance inside the host lies in the evasion of cellular intrinsic and host immune defenses. As such, molecular warfare has driven evolution to enable co-speciation of the herpesviruses with their individual mammalian hosts over millions of years. A unique adaptation of the gammaherpesvirus subfamily (γHVs) is the capture and repurposing of the cellular purine metabolism enzyme, formyl-glycinamide-phosphoribosyl-amidotransferase (FGARAT) to support infection [1–3]. The human herpesviruses Epstein-Barr virus (EBV/HHV-4) and Kaposi’s sarcoma-associated herpesvirus (KSHV/HHV-8) each encode a single viral FGARAT (vFGARAT), yet other gammaherpesviruses encode multiple vFGARATs [1]. The primate rhadinovirus herpesvirus saimiri (HVS) encodes two vFGARATs with distinct functions [4], and the murine gammaherpesviruses have invested ~10% of their genomes to encode three vFGARATs, *orf75A*, *orf75B*, and *orf75C*.

The vFGARAT proteins examined to date are localized within the tegument of virions and are delivered to newly infected cells to immediately act on the cell [5–8]. Nuclear structures termed PML-associated nuclear bodies (PML-NBs, also known as ND10s or PODs) serve as a first line of defense and house an arsenal of antiviral factors such as PML, Sp100, Daxx, and ATRX [9]. Each vFGARAT impairs the function of a component of PML-NBs, albeit via different mechanisms such as relocalization or degradation. MHV68 ORF75C degrades PML [10]. EBV BNRF1 interacts with Daxx to dissociate it from ATRX to effectively neutralize gene silencing of the viral genome [11, 12], while KSHV ORF75 targets ATRX for degradation and relocalizes the other major PML-NB components [3]. The consequences of vFGARAT interactions with host PML-NBs are not completely understood. MHV68 ORF75C is required for replication, but the degradation of PML by ORF75C is not likely the essential function [13].

vFGARATs are large ~148 kDa proteins with multiple functions that appear to impact early and late stages of infection. MHV68 ORF75C and KSHV ORF75 interact with the host FGARAT (*PFAS*) leading to the non-canonical deamidation of RIG-I by the host FGARAT to drive NF-κB signaling [3, 10, 14]. Unlike MHV68 ORF75C, exogenous expression of MHV68 ORF75A or ORF75B does not lead to PML relocalization or degradation, and these proteins do not drive RIG-I deamidation [10, 14]. Moreover, MHV68 infection does not result in Sp100 degradation in human fibroblasts [4] or Daxx degradation in human [4] or mouse fibroblasts [10]. Thus, degradation of PML-NB components are unlikely functions of either MHV68 ORF75A or ORF75B. The consequences of vFGARAT disruption on pathogenesis in the host have not been reported.

Here, we set out to identify the roles of the less well-characterized vFGARATs of the murine rhadinoviruses, ORF75A and ORF75B, during viral replication in cell culture and pathogenesis in the host. To do so, we generated and characterized individual 75A.stop and 75B.stop mutant viruses. We find that loss of ORF75B did not impair replication in cell culture or in mice, suggesting functional overlap with ORF75A or ORF75C. In contrast, loss of ORF75A led to a significant defect in the production of particles capable of plaque formation. This replication defect had several manifestations *in vivo*, including the failure to expand during acute infection of the lung and to colonize multiple lymphoid organs with wild-type kinetics, and compromised reactivation from latency in the spleen. Our data indicate that the duplicated vFGARATs in the murid gammaherpesviruses have diverged such that ORF75A has separable functions from ORF75C that are required for pathogenesis in the host.

## Results

### Evolutionary divergence within the vFGARATs

**Fig 1:**
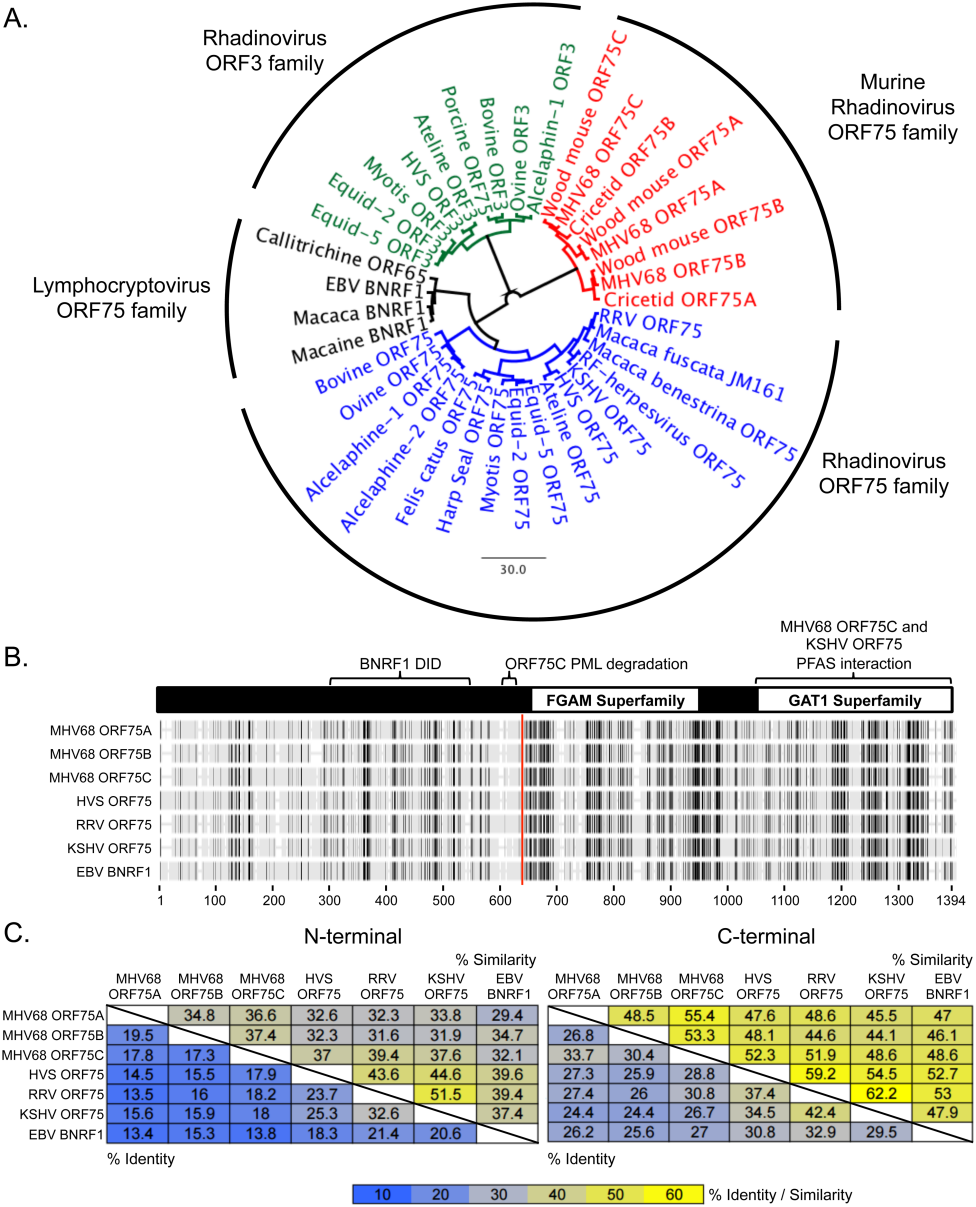
Divergence of the gammaherpesvirus vFGARAT family. **(A)** Phylogenetic tree of the vFGARATs of the indicated γ-hepesviruses (Genebank ID located in S1 Table). **(B)** Phylogenetic alignment using BLOSUM62 with previously characterized domains of the vFGARATs. **(C)** Pairwise alignment showing degrees of identity (lower left corner of table) or similarity (upper right corner of table) between the respective N-terminal or C-terminal portions of the indicated vFGARATs. The division point for each vFGARAT (red vertical line in B) was based on dotplot analysis for each indicated vFGARATS compared to MHV68 ORF75C.

All gammaherpesviruses encode at least one tegument protein that shares amino acid similarity with the host cellular purine metabolism enzyme formyl-glycinamide-phosphoribosyl-amidotransferase (FGARAT). The rhadinoviruses of rodents encode two or three vFGARATs, and the primate rhadinovirus herpesvirus saimiri (HVS) encodes two vFGARATs. We performed a phylogenetic analysis of the amino acid sequences of the vFGARATs, and identified four major clades across multiple species within the lymphocryptovirus and rhadinovirus subfamilies: lymphocryptovirus ORF75, rhadinovirus ORF3, rhadinovirus ORF75, and murine rhadinovirus ORF75 homologs (Fig 1A). The placement of the rhadinovirus ORF3 and ORF75 homologs into unique clades is consistent with distinct functions during infection [4].

The MHV68 ORF75 homologs share an overall ~25% amino acid sequence identity among themselves and with other primate homologs. Alignment of the MHV68 proteins with vFGARATs from other primate gammaherpesviruses reveals a C-terminus with large regions of homology within the FGAM and GAT1 superfamily domains. The C-terminal portion of ORF75C and KSHV ORF75, but not EBV BNRF1, are sufficient to interact with the human FGARAT (PFAS) [14]. However, domains required for two divergent vFGARAT functions, interaction of EBV BNRF1 with host Daxx [11] and degradation of host PML by MHV68 ORF75C [15], lie within the N-terminus (Fig 1B). In separate pairwise alignments for the N-terminal and C-terminal domains, the primate and murine gammaherpesviruses have a higher degree of sequence conservation to members within, rather than outside, their species (Fig 1C). Each murine rhadinovirus vFGARAT is equidistant from the human gammaherpesvirus homologs, and led us to examine what unique roles the murine vFGARATs ORF75A and ORF75B might provide for viral infection and pathogenesis.

### Differential impact of disruption of ORF75A or ORF75B on MHV68 replication

**Fig 2:**
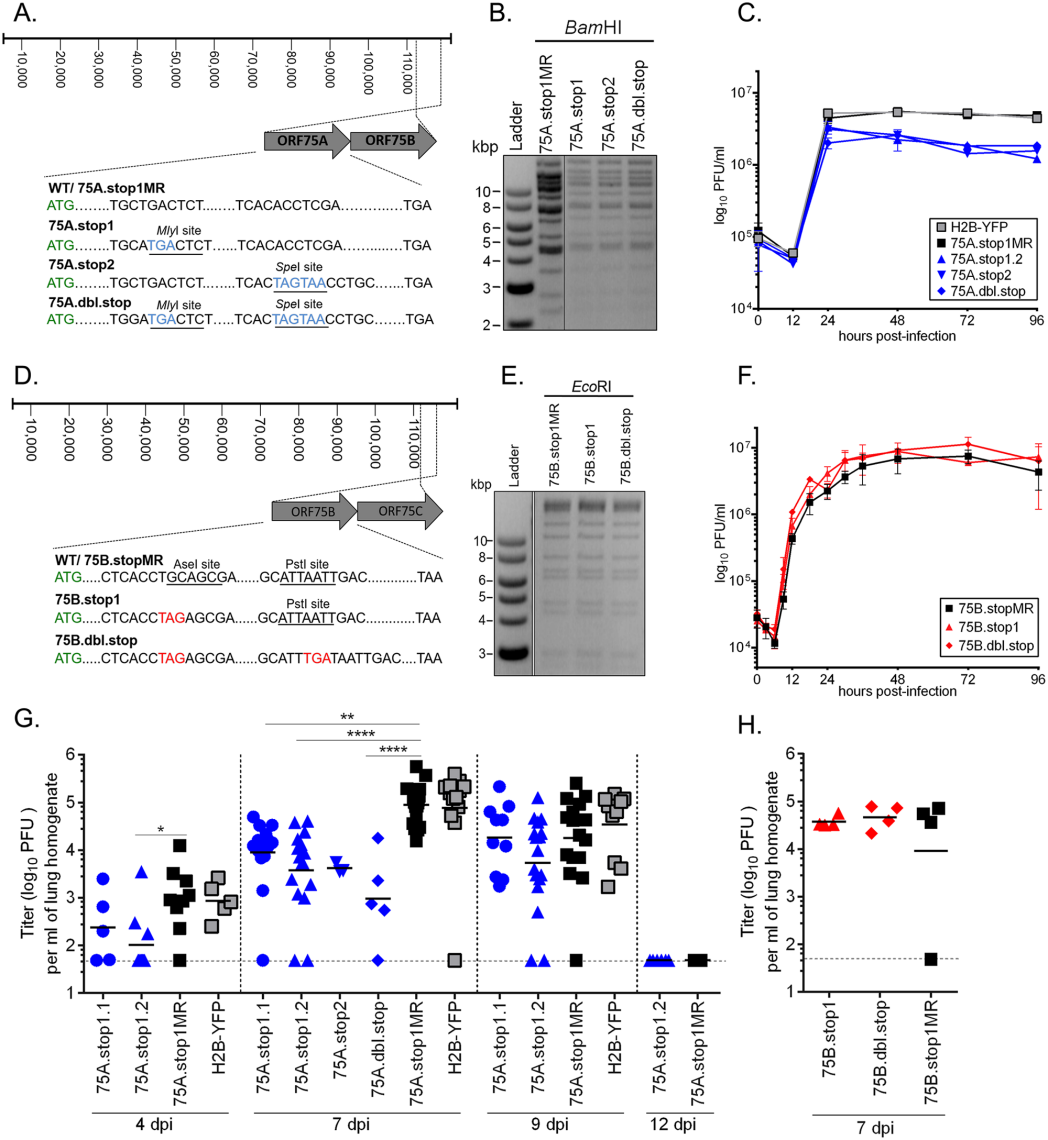
Generation and characterization of 75A.stop and ORF75B.stop viruses. **(A)** Schematic of the 75A.stop mutations in MHV68. Restriction sites used to verify stop codon insertions are underlined. **(B)** Restriction fragment length polymorphism (RFLP) analysis of 75A.stop mutants after BamHI digestion. **(C)** Single-step growth curve of 75A.stop mutants and parental H2B-YFP viruses in NIH 3T3 fibroblasts (MOI 5). **(D)** Schematic of the ORF75B mutations in MHV68. **(E)** RFLP analysis of ORF75B.stop mutants after EcoRI digestion. **(F)** Single-step growth of ORF75B.stop mutants and marker rescue virus in NIH 3T3 fibroblasts (MOI 5). Error bars represent +/- SD of triplicate infections. C57B/6 mice were inoculated with 1000 PFU via the intranasal route with the indicated viruses. Lung homogenates from mice were titered by plaque assay. **(G)** Timecourse analysis of acute replication on the lungs. **(H)** Acute replication in the lungs 7 dpi. Each symbol represents an individual mouse. Line indicates geometric mean titer. The dashed line depicts the limit of detection at 50 PFU/ml of lung homogenate (log_10_ of 1.7). * p ≤ 0.05; ** p ≤ 0.005; **** p ≤ 0.0005; for G-H, significance determined by one-way ANOVA analysis.

In a previous screen of a MHV68 transposon-mutant library, replication defects were reported for mutants with a transposon insertion in ORF75A [16]. Since a large 1.2 kb transposon insertion may impact neighboring genes or antisense transcripts [17, 18], we generated new recombinant viruses with insertions of a smaller all-frames stop codon in either *orf75A* or *orf75B* downstream of the first methionine (101 aa and 78 aa, respectively) (Fig 2A and 2D). These proximal stop codon disrupted viruses are herein termed 75A.stop1 (with independent isolates termed 75Astop1.1 and 75Astop1.2) and 75B.stop1. A second set of ORF75A mutant viruses (75A.stop2) were constructed with the stop codon insertions at the same position (aa 428) as the previously reported transposon disruptions [16]. Finally, we generated viruses harboring double-stop codons in either *orf75A* (101 aa and 428 aa) or *orf75B* (78 aa and 351 aa) (75A.dbl.stop or 75B.dbl.stop, respectively). We repaired the proximal 75A.stop1 BAC and ORF75B.stop1 BAC back to wild-type sequence (75A.stop1MR and 75B.stop1MR, respectively) (Fig 2A and 2D). All recombinants were generated on the MHV68-H2BYFP marking virus BAC platform [19, 20]. Restriction fragment length polymorphism (RFLP) analysis (Fig 2B and 2E) and whole genome sequencing confirmed that there were no additional mutations in the mutant and marker rescue viruses.

To examine the impact of the 75A.stop mutations on MHV68 replication, we measured viral growth in immortalized fibroblasts (NIH 3T3) following infection at a MOI of 5. In these single-step growth curves, each 75A.stop virus replicated with similar kinetics compared to the parental MHV68-H2BYFP and 75A.stop1MR virus, yet exhibited a two to three-fold decrease in virus production (Fig 2C). 75B.stop1 and 75B.dbl.stop virus growth kinetics were indistinguishable from the 75B.stop1MR virus (Fig 2F). Thus, in contrast to the replication-essential ORF75C gene, viruses lacking ORF75A were attenuated for peak virus production while ORF75B was dispensable for replication in immortalized fibroblasts at a high multiplicity of infection.

We next examined acute replication in the lungs of mice upon intranasal infection with 1000 PFU of the mutant viruses compared to control viruses. At 4 days post infection (dpi), 75A.stop1 virus replication was only detected in the three of the five mice. By 7 dpi, 75A.stop1MR virus replication had increased by two orders of magnitude. In contrast, mean titers from the lungs of mice infected with either of the two independent isolates of 75A.stop1 (75A.stop1.1 and 75A.stop1.2), 75A.stop2, or 75A.dbl.stop were significantly lower than 75A.stop1MR, ranging between a one to two log reduction in mean titer. By 9 dpi, titers of 75A.stop1 and 75A.stop1MR were more comparable, and by 12 dpi the acute lung infection was resolved (Fig 2G). In contrast to the ORF75A mutants, the 75B.stop1 and 75B.dbl.stop viruses replicated to similar levels as the 75B.stop1MR viruses at 7 dpi (Fig 2H). This data is consistent with previous reports for ORF75A and ORF75B mutants with a transposon insertion [16]. Thus, ORF75A promotes replication in the lungs of infected mice while ORF75B is dispensable for acute lung replication.

### ORF75A promotes the establishment of latency in multiple reservoirs upon intranasal inoculation

**Fig 3:**
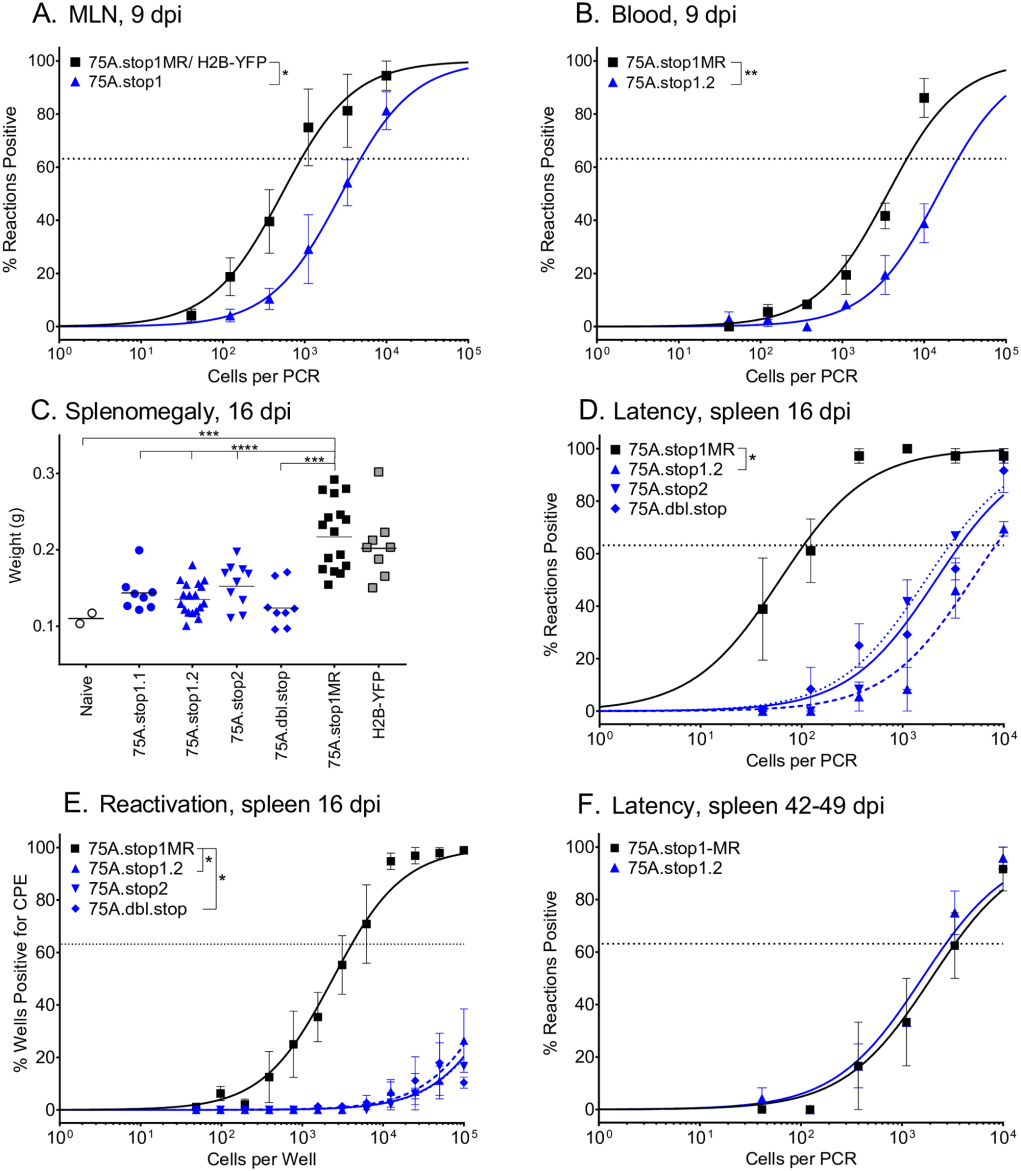
MHV68 ORF75A is essential for efficient seeding of intermediate reservoirs and the establishment of latency in the spleen at early, but not late times of chronic infection after intranasal inoculation. C57BL/6 mice were infected at 1000 PFU by the intranasal route with the indicated viruses. **(A)** Frequency of cells in the mediastinal lymph node harboring viral genomes 9 dpi. Data is a compilation of 75A.stop1.1 (n = 1) with 75A.stop1.2 (n = 4) and of H2BYFP (n = 1) with 75A.stop1MR (n = 4). **(B)** Frequency of peripheral blood mononuclear cells harboring genomes 9 dpi. Data is generated from 3 independent experiments, 5 mice per group. **(C)** Splenomegaly 16 dpi with indicated viruses. Each symbol represents an individual mouse. Line indicates geometric mean titer. ORF75A mutants are blue and WT control viruses are black. **(D)** Frequency of splenocytes harboring genomes 16 dpi. **(E)** Frequency of splenocytes undergoing reactivation from latency upon explant 16 dpi. **(F)** Frequency of splenocytes harboring genomes at six weeks post-infection. For the limiting dilution analyses, curve fit lines were determined by nonlinear regression analysis. Using Poisson analysis, the intersection of the nonlinear regression curves with the dashed line at 63.2% was used to determine the frequency of cells that were either positive for the viral genome or reactivating virus. **(C-F)** Data is generated from two independent experiments for 75A.stop2 and 75a.dbl.stop viruses, and three independent experiments for 75A.stop1.2 and 75A.stop1MR viruses. Each experiment contained three to six mice per group for 16 dpi. Data for 42–49 dpi is generated from two experiments with four to five mice per group. Error bars indicate SEM. * p ≤ 0.05, *** p ≤ 0.0005, and **** p ≤ 0.00005; for A, B, D-F, significance determined by two-way paired t-test; for C, significance determined by two-way unpaired t-test.

Prior to and concomitant with colonization of the spleen, MHV68 is detected both in the mediastinal lymph nodes (MLN) that survey the lymph exiting the lungs and in peripheral blood mononuclear cells (PBMCs) [21–24]. Given the severity of acute replication in the lungs, we investigated the ability of 75A.stop viruses to infect these intermediate reservoirs. The absence of ORF75A resulted in a five-fold reduction in the frequency of cells of the MLN infected with MHV68 at 9 dpi (Fig 3A, **summarized in** Table 1). The frequency of PBMC infection was decreased four-fold for 75A.stop1 compared to the 75A.stop1MR virus (Fig 3B, Table 1).

**Table 1:** Frequencies of cells harboring viral genomes in C57BL/6 mice.

Virus^a^	Route of infection^b^	Organ^c^	dpi	Total # of cells harvested	Frequency of genome-positive cells (one in X cells) ^d^	Total # of cells positive for latent virus ^e^
**75A.stop1MR**	i.n.	MLN	9	1.2 x10^7^	907	1.3 x10^4^
**75A.stop1.2**	i.n.	MLN	9	1.1 x10^7^	4,833	2.3 x10^3^
**75A.stop1MR**	i.n.	Blood	9	4.2 x10^5^	6,088	6.9 x10^1^
**75A.stop1.2**	i.n.	Blood	9	3.3 x10^5^	25,765	1.3 x10^1^
**75A.stop1MR**	i.n.	Spleen	16	9.1 x10^8^	104	8.8 x10^6^
			42	7.5 x10^8^	1,760	4.3 x10^5^
**75A.stop1.2**	i.n.	Spleen	16	3.6 x10^8^	8,362	4.3 x10^4^
			42	7.5 x10^8^	2,282	3.3 x10^5^
**75A.stop2**	i.n.	Spleen	16	0.6 x10^9^	2,875	2.1 x10^5^
**75A.dbl.stop**	i.n.	Spleen	16	0.7 x10^9^	3,622	1.9 x10^5^
**75A.stop1MR**	i.p.	Spleen	18	5.0 x10^8^	278	1.8 x10^6^
			46	4.3 x10^8^	5,269	8.2 x10^4^
**75A.stop1.2**	i.p.	Spleen	18	3.6 x10^8^	588	6.1 x10^5^
			46	4.1 x10^8^	4,561	9.0 x10^4^
**75A.stop1MR**	i.p.	PEC	18	4.4 x10^7^	55	8.0 x10^5^
**75A.stop1.2**	i.p.	PEC	18	3.5 x10^7^	85	4.1 x10^5^
**75B.stop1**	i.n.	Spleen	16	1.3 x10^9^	147	8.8 x10^6^
**75B.dbl.stop**	i.n.	Spleen	16	0.9 x10^9^	404	2.2 x10^6^
**75B.stop1MR**	i.n.	Spleen	16	1.5 x10^9^	123	1.2 x10^7^

^a^ Infection with recombinant MHV68 viruses

^b^ i.n., intranasal; i.p., intraperitoneal

^c^ Organ harvested for limiting dilution analysis (MLN, mediastinal lymph node PBMC, peripheral blood mononuclear cell)

^d^ The frequency data were determined from the mean of two to five independent experiments with cells from the indicated organs. Organs were pooled from three to five mice per experiment.

^e^ The total number of genome positive cells per mouse was extrapolated using the frequency value generated from the limiting dilution analysis together with the total number of splenocytes or PEC cells harvested.

Within two weeks of intranasal infection with MHV68, virus replication in the lung is resolved and the virus transits to the spleen where it establishes latency, predominantly in B lymphocytes [22, 25, 26]. Peak splenic latency occurs between 14 and 18 dpi and coincides with splenomegaly, a process driven by lymphocyte expansion in response to the infection [27]. The spleen weights of mice infected with the 75A.stop1, 75A.stop2, or 75A.dbl.stop viruses were only slightly larger than those of naïve mice at 16 dpi, and exhibited a two-fold decrease in splenomegaly compared to 75A.stop1MR-infected mice (Fig 3C). This defect in splenomegaly correlated with a severe two-log reduction in the frequency of splenocytes harboring the viral genome in mice infected with the 75A.stop viruses as compared to 75A.stop1MR (Fig 3D, Table 1). This impairment in latency establishment was accompanied by a similar reduction in the frequency of reactivation from latency upon explant (Fig 3E, **summarized in** Table 2). The severity of the defect in the establishment of latency precluded a determination of whether ORF75A plays a role in splenic reactivation. Despite a severe latency defect at 16 dpi, the frequency of genome-positive splenocytes six weeks after infection with the 75A.stop1 virus was nearly equivalent to 75A.stop1MR (Fig 3F, Table 1). These data indicate a critical role for ORF75A in efficiently seeding the intermediate reservoirs and initial establishment of latency, but not long-term establishment and maintenance of latency after intranasal inoculation of mice.

**Table 2:** Frequencies of cell populations reactivating viral genomes in C57BL/6 mice.

Virus^a^	Route of infection^b^	Organ^c^	dpi	Total # of cells harvested	Frequency of genome-positive cells (one in x cells) ^d^	Total # of cells reactivating latent virus ^e^
**75A.stop1MR**	i.n.	Spleen	16	9.1 x10^8^	4.1 x 10^3^	2.2 x 10^5^
**75A.stop1.2**	i.n.	Spleen	16	3.6 x10^8^	5.2 x 10^5^	6.9 x 10^2^
**75A.stop2**	i.n.	Spleen	16	0.6 x10^9^	6.9 x 10^5^	8.7 x 10^2^
**75A.dbl.stop**	i.n.	Spleen	16	0.7 x10^9^	6.7 x 10^5^	1.0 x 10^3^
**75A.stop1MR**	i.p.	Spleen	18	5.0 x10^8^	2.0 x10^4^	2.5 x 10^4^
**75A.stop1.2**	i.p.	Spleen	18	3.6 x10^8^	1.0 x10^5^	3.6 x 10^3^
**75A.stop1MR**	i.p.	PEC	18	4.4 x10^7^	3.0x10^3^	1.5 x 10^4^
**75A.stop1.2**	i.p.	PEC	18	3.5 x10^7^	2.1x10^3^	1.6 x 10^4^
**75B.stop1**	i.n.	Spleen	16	1.3 x10^9^	6.1x10^3^	2.1 x 10^5^
**75B.dbl.stop**	i.n.	Spleen	16	0.9 x10^9^	6.8x10^3^	1.3 x 10^5^
**75B.stop1MR**	i.n.	Spleen	16	1.5 x10^9^	6.8x10^3^	2.2 x 10^5^

^a^ Infection with recombinant MHV68 viruses

^b^ i.n., intranasal; i.p., intraperitoneal

^c^ Organ harvested for limiting dilution analysis (MLN, mediastinal lymph node PBMC, peripheral blood mononuclear cell)

^d^ The frequency data were determined from the mean of two to five independent experiments with cells from the indicated organs. Organs were pooled from three to five mice per experiment.

^e^ The total number of cells reactivating latent virus per mouse was extrapolated using the frequency value generated from the limiting dilution analysis together with the total number of splenocytes or PEC cells harvested.

In contrast to the latency defect observed with the 75A.stop viruses, intranasal inoculation of mice with either 75B.stop1 or 75B.dbl.stop did not impact latency establishment. We observed no difference in the frequency of cells harboring MHV68 genomes between 75B.stop1, 75B.dbl.stop, and 75B.stop1MR infections (S1A Fig, Table 1). Moreover, loss of MHV68 ORF75B had no impact on the frequency of reactivation from splenic latency (S1B Fig, Table 2). Thus, in addition to the lack of a replication defect in the lung during acute phase of infection, we did not observe a role for ORF75B in splenic latency or reactivation after intranasal infection.

### Bypassing the lung reveals a role for ORF75A in promoting reactivation from latency

**Fig 4:**
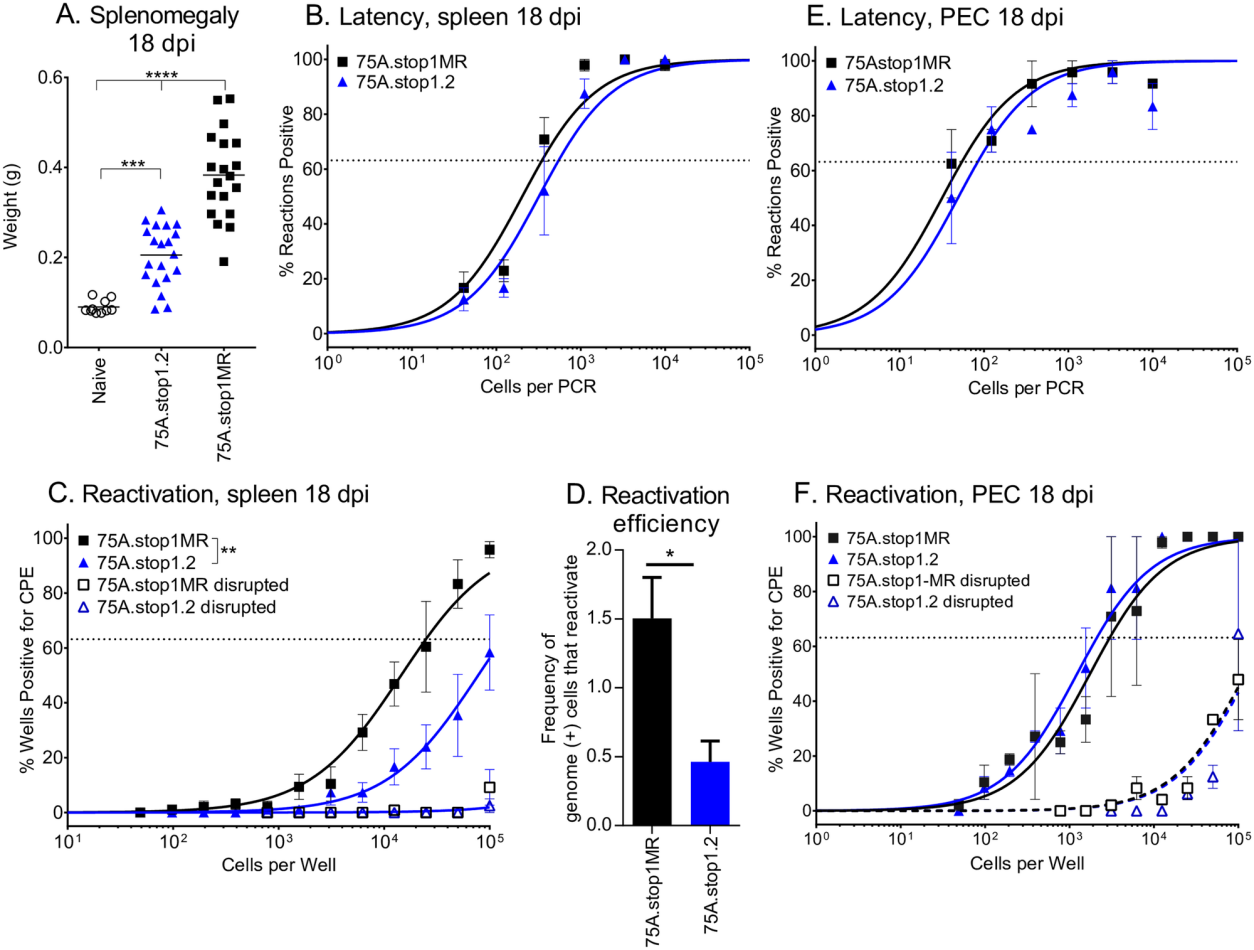
Intraperitoneal administration of MHV68 reveals a reactivation defect in the absence of ORF75A. C57BL/6 mice were infected at 1000 PFU by the intraperitoneal route with the indicated viruses. **(A)** Splenomegaly at 18 dpi with indicated viruses. Each symbol represents an individual mouse. Line indicates geometric mean titer. **(B)** Frequency of splenocytes harboring genomes 18 dpi. **(C)** Frequency of splenocytes spontaneously reactivating from latency 18 dpi. **(D)** Reactivation efficiency of splenocytes 18 dpi. **(E)** Frequency of PECs harboring genomes 18 dpi. **(F)** Frequency of PECs spontaneously reactivating from latency 18 dpi. For the limiting dilution analyses, curve fit lines were determined by nonlinear regression analysis. Using Poisson analysis, the intersection of the nonlinear regression curves with the dashed line at 63.2% was used to determine the frequency of cells that were either positive for the viral genome or reactivating virus. Data is generated from 5 independent experiments with 4–5 mice per group for splenocyte data and 2 independent experiments with 4 mice per group for PEC data. ** p ≤ 0.005, *** p ≤ 0.0005, and **** p ≤ 0.00005; for A significance determined by two-way unpaired t-test; for B-F significance determined by two-way paired t-test.

The intranasal route identified a critical role for ORF75A in lung replication and multiple latency reservoirs, including the MLN and PBMCs prior to seeding the spleen. To bypass the restriction of the lungs and determine if ORF75A has a role in latency establishment or reactivation in the spleen independent of its impact on dissemination to that organ, we administered the virus by intraperitoneal (IP) inoculation. Splenomegaly was reduced in mice infected with 75A.stop viruses (Fig 4A), but there was not a significant decrease in the frequency of splenic cells that harbored the viral genome upon infection with 75A.stop1 as compared to 75A.stop1MR at 16 dpi (Fig 4B, Table 1) and 42 dpi (S2 Fig, Table 1). This indicates that ORF75A is not required to establish latency in splenocytes when the virus is administered by a more direct route. However, there was a significant decrease in the frequency of cells that reactivated from latency (Fig 4C, Table 2) following infection with 75A.stop1 compared to 75A.stop1MR (Fig 4D).

In contrast, loss of ORF75A did not influence either the frequency of genome positive peritoneal exudate cells (PEC) or viral reactivation from this compartment upon explant (Fig 4E and 4F, Tables 1 and 2). Thus, the IP route of administration revealed a cell type-specific requirement for ORF75A in efficient viral reactivation from latently infected splenocytes, but not PECs.

### Loss of ORF75A alters virus gene expression and reduces replication in primary cells

We next characterized the expression of ORF75A and its impact on productive infection in cell culture. ORF75A was previously identified as a virion protein that is present in the nucleus of infected cells [1]. We generated a recombinant MHV68 with a 3X-FLAG epitope tag fused to the N-terminus of ORF75A (referred to as FLAG-75A) (S3A Fig) that did not disrupt replication (S3B Fig), but enabled detection of the expected ~145 kDa fusion protein by immunoblot in MEFs (MOI 5) nine hours post infection (hpi) (S3C Fig). FLAG-75A was not detected in NIH 3T3 cells by immunofluorescence, consistent with the observed low level of expression by immunoblot (S3C Fig) and previous reports of low *orf75A* transcript levels [18]. This led us to examine the localization of CMV-IE promoter driven FLAG-ORF75A upon transfection or coupled with MHV68-H2BYFP infection in NIH 3T3 cells. FLAG-ORF75A was predominately localized to the nucleus (S3D Fig, **top**), but in the majority of infected cells expressing YFP, nuclear FLAG-ORF75A was reduced (S3D Fig, **bottom**).

**Fig 5:**
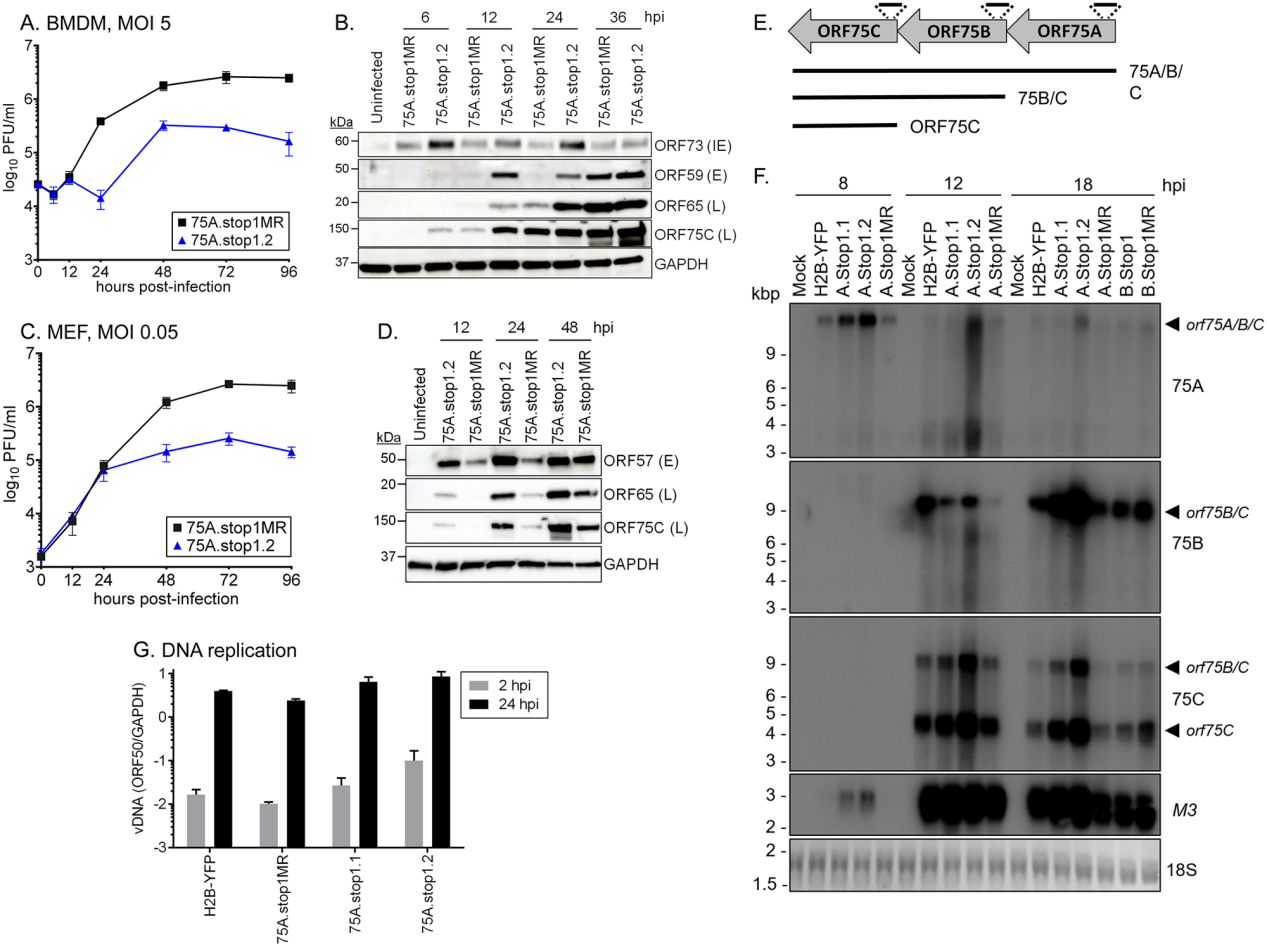
Accelerated gene expression coupled with replication defect upon 75A.stop infection of primary cells. **(A)** Single-step growth curve in primary BMDM at an MOI of 5 with 75A.stop1.2 and 75A.stop1MR. **(B)** Timecourse analysis of gene products upon a single-step infection of BMDMs. **(C)** Multi-step growth curve in primary MEFs at an MOI of 0.05 with 75A.stop1.2 and 75A.stop1MR. **(D)** Timecourse analysis of gene products upon a low MOI infection of MEFs. **(E)** Schematic of the ORF75 locus and the multicistronic transcripts with strand-specific probes to indicated ORFs. **(F)** Northern blot analysis of NIH 3T12 fibroblast cells infected with indicated viruses at an MOI of 5. Membrane was hybridized with strand-specific ^32^P-labled cDNA of ORF75A, ORF75B, ORF75C, M3, or 18S. Longer exposures with ORF75C probe detects the *orf75A/B/C* transcript. **(G)** Viral DNA quantification of input genomes at 2 hpi and subsequent replication at 24 hpi. For A, C, and E, bars represent the mean of three independent biological replicates +/- SD.

We next examined 75A.stop1 and 75A.stop1MR replication in more restrictive cell culture conditions. We infected primary bone marrow derived macrophages (BMDMs) with 75A.stop1 or 75A.stop1MR viruses at a MOI of 5, which generally results in ~30% initial infection. The kinetics of 75A.stop1 and 75A.stop1MR replication were comparable for the first 12 hrs of infection (Fig 5A). The 75A.stop1MR control virus continued exponential growth from 12 to 24 hpi, reaching a plateau between 72 and 96 hpi. In contrast, the 75A.stop1 virus had a dramatic one-log reduction in replication characterized by an early plateau of infectious particle production (Fig 5A).

In light of the growth defect observed with 75A.stop1, we examined the effect of ORF75A loss on the expression of viral proteins, including those of the late kinetic class that generally drive virion assembly and release. Surprisingly, viral protein expression from all kinetic classes was elevated in 75A.stop1-infected cells as compared to 75A.stop1MR-infected cells. Specifically, we observed higher levels of immediate early (IE) ORF73 at 6 hpi, early (E) protein ORF59 at 12 and 24 hpi, and the late (L) capsid protein ORF65 at 12 and 24 hpi. ORF75C, a tegument protein with classical late gene kinetics [15], exhibited a striking increase in protein levels following infection with 75A.stop1 compared to the 75A.stop1MR at 6 hpi (Fig 5B).

In the absence of ORF75A, virus replication also prematurely plateaued in primary MEFs (MOI 0.05). 75A.stop1 replication peaked at 72 hpi, while 75A.stop1MR continued to increase by an additional log between 48 and 96 hpi (Fig 5C). Again, we observed a noticeable increase in viral protein levels across each kinetic class, including IE ORF57, L ORF65 and ORF75C, at each timepoint (Fig 5D). In addition we observe a similar, albeit lesser, defect in virus production and altered kinetics of viral protein expression in a single-step growth curve in MEFs (MOI 5.0) (S4 Fig).

All kinetic classes of viral proteins, including tegument ORF75C, were detected at higher levels throughout infection with 75A.stop1 (Fig 5B and 5D). Thus, we assessed the structure and levels of vFGARAT transcripts by northern blot analysis (Fig 5E and 5F). A single polyadenylation signal proximal to the 3’ end of *orf75C* predicted that transcription of the ORF75 locus would proceed via a co-terminal, multicistronic transcript. Strand-specific probes to *orf75A* (S3 Table) detected low-levels of a large transcript by 8 hpi that was consistent with the expected 12 kb *orf75A/B/C* polycistronic RNA (Fig 5E). Upon longer exposure, the ORF75C probe detected the *orf75A/B/*C transcript (S5 Fig). The ORF75B andORF75C probes detected an ~ 8 kb transcript at 12 hpi, suggestive of an *orf75B/C* bicistronic message (Fig 5F). A single ~4 kb *orf75C* transcript was detected with the ORF75C probe (Fig 5F). Consistent with the polyA site and predictions from microarray analyses [17, 18], transcription of the MHV68 *orf75* locus appears to be characterized by staggered initiation of transcription generating overlapping multicistronic messages. Notably, the stop codon insertions in *orf75A* or *orf75B* did not alter the size of the *orf75* transcripts. However, all transcripts examined, including the internal control lytic gene *M3*, were elevated following infection with 75A.stop1 (Fig 5F).

This increase in expression of multiple lytic genes upon 75A.stop infection led us to investigate whether viral DNA replication was enhanced. Primary MEFs were infected with 75A.stop1MR or 75A.stop1 viruses, followed by a citric acid wash to remove extracellular virus. We observe a notable increase in the levels of viral DNA at 2 hpi in the 75A.stop samples. This indicated that more viral genomes were delivered to the cells infected with 75A.stop compared to either parental MHV68-H2BYFP or 75A.stop1MR viruses, and that difference is maintained at 24 hpi. However, when the viral genome copy number at 24 hpi was normalized to the input genomes within the cells at 2 hpi there was a similar ~150 to 200-fold increase in viral genome copy number for all virus infections between 2 and 24 hpi (Fig 5G). Thus, loss of ORF75A did not enhance viral DNA synthesis. Taken together, the loss of ORF75A has severe consequences for virus replication including increased delivery of viral genomes, dysregulated viral gene expression, and decreased infectious particle production.

### Virus particles generated in the absence of ORF75A are reduced in plaque formation

**Fig 6:**
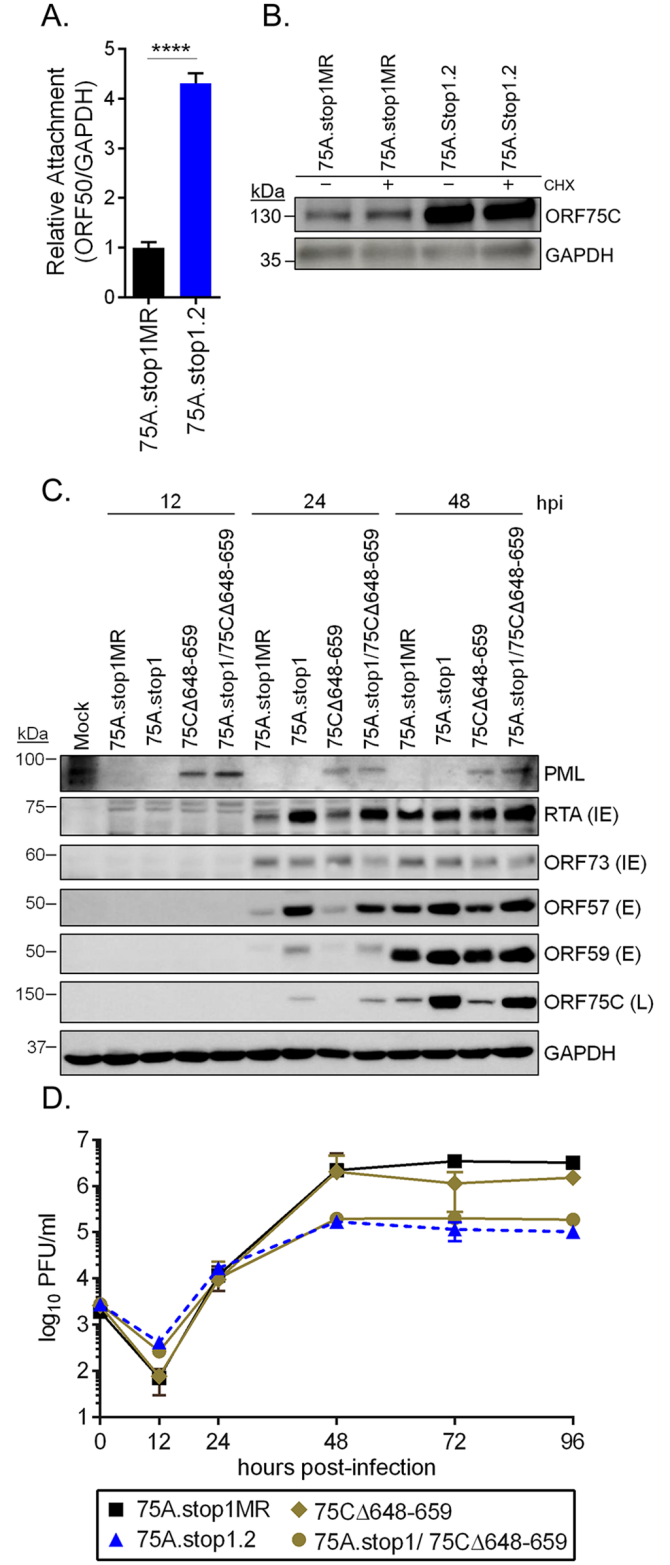
PML degradation by ORF75C tegument does not contribute to 75A.stop replication defect. **(A)** Analysis of viral attachment. Primary MEFs were infected at an MOI of 0.05 with the indicated viruses at 4°C to inhibit virus entry. **** p ≤ 0.00005; significance determined by two-way unpaired t-test. **(B)** Immunoblot analysis of tegument-derived ORF75C ± cycloheximide (CHX) treatment upon infection with indicated viruses (MOI 5.0). **(C)** Immunoblot analysis of PML degradation kinetics and viral protein expression upon a multi-step infection (MOI 0.05) with indicated viruses. **(D)** Multi-step growth curve in primary MEFs with indicated viruses (MOI 0.05). Error bars represent the mean of three independent biological replicates ± SD.

75A.stop1 virus infection led to both an increase in ORF75C protein levels at early timepoints and the delivery of more viral genomes, suggesting that more 75A.stop virions were bound and entered the infected cells compared to the revertant virus. To examine attachment, we infected MEFs for 90 minutes at 4°C with 75A.stop1.2 or marker rescue virus at equivalent MOI followed by two cold PBS washes. Viral DNA was extracted from the samples and quantified via qPCR. Indeed, there was a four-fold increase in 75A.stop1.2 virus attachment compared to MR virus (Fig 6A). We next investigated if these virions enter and release their tegument into the infected cell. To do so, we examined the deposition of ORF75C, a major component of the tegument [5, 6] into NIH 3T12 cells that were treated with cycloheximide prior to, and during, infection. At 2 hpi, ORF75C was detected at higher levels in cultures infected with 75A.stop1 virus compared to 75A.stop1MR virus, indicating that the presence of ORF75C protein in newly infected cells was independent of de novo protein synthesis (Fig 6B). In addition, we observed a similar increase in ORF75C deposition in newly infected BMDMs (S6 Fig). These data indicate that the increased levels of ORF75C detected in the ORF75A stop infections is derived from the tegument of incoming virions.

Multiple studies have identified roles for PML-NBs in the transcriptional repression of herpesvirus genomes [28–34]. Depletion of PML enhanced the replication of an ICPO-null HSV-1 [30, 35] and HCMV [34], and the replication and reactivation of MHV68 [13, 36]. ORF75C promotes the degradation of PML via a putative E3-ubiquitin ligase function [1, 10]. Thus, the enhanced lytic gene expression upon 75A.stop1 infection might be attributed to an increased deposition of tegument-ORF75C. Sewatanon et al. [13] characterized an ORF75C mutant (75CΔ648–659) that cannot degrade PML. To test if our observed ORF75A phenotype is dependent on PML degradation by ORF75C, we generated a combinatorial mutant virus with the 75A.stop1 mutation and a deletion of amino acids 648–659 in ORF75C, and generated the single mutant 75CΔ648–659 virus as a control. As previously reported, the ORF75CΔ648–659 mutation ablated PML degradation upon infection of primary fibroblasts, but this did not reduce viral protein expression (Fig 6C) or replication (Fig 6D) of the single 75CΔ648–649 mutant. Moreover, the enhanced viral protein expression upon infection with the combinatorial 75A.stop1/75CΔ648–649 mutant was comparable to 75A.stop (Fig 6C). In addition, the combinatorial 75A.stop1/75CΔ648–649 mutant retained the replication defect of the single 75A.stop mutant (Fig 6D). Thus, the 75A.stop phenotype is independent from the PML degradation function of ORF75C.

**Fig 7:**
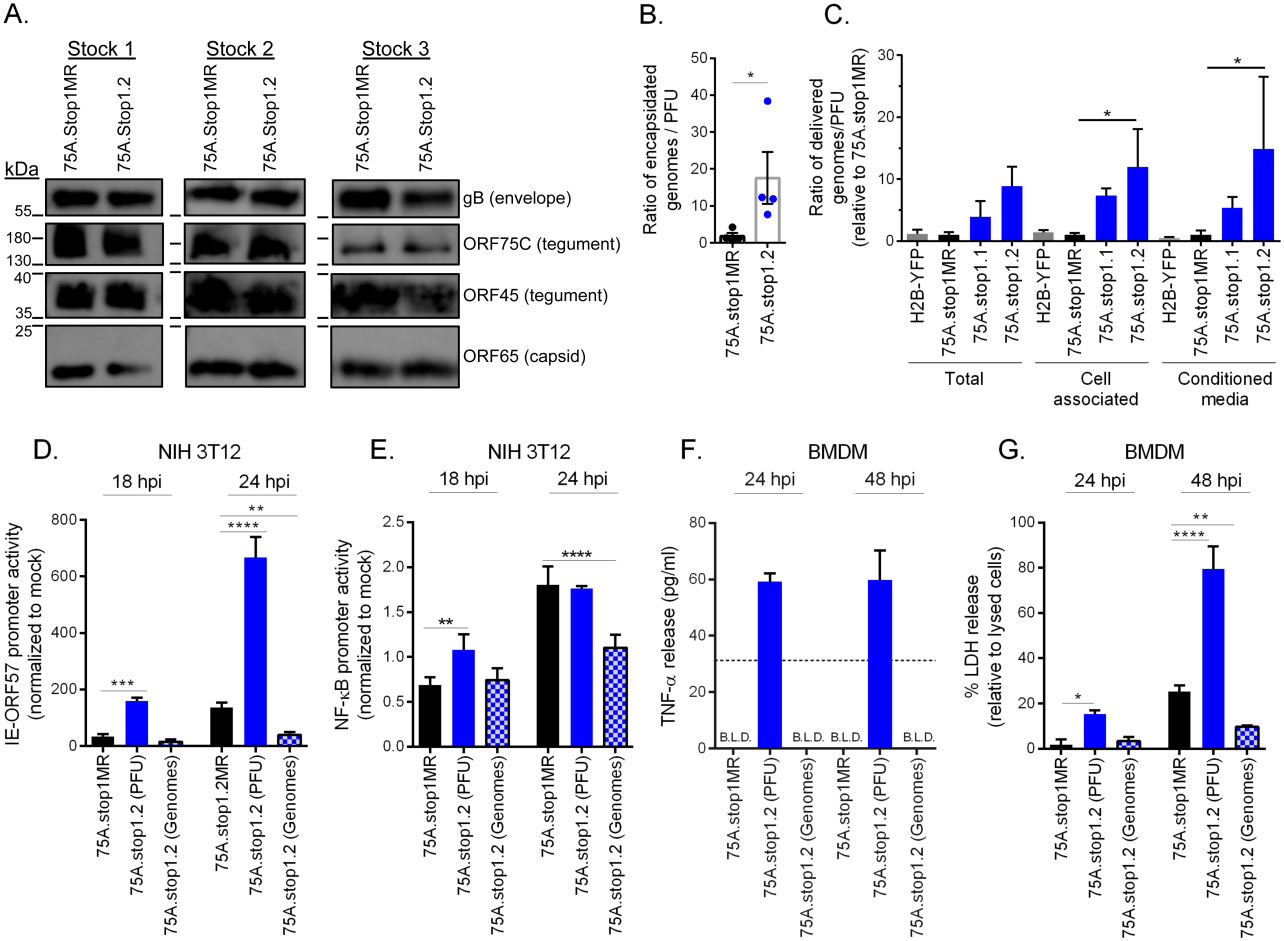
Input of equivalent genomes ameliorates the high particle to PFU phenotype of 75A.stop. **(A)** Immunoblot analysis of purified virions from three independent virus stock preparations. **(B)** Quantification of the particle to PFU ratio. Particles were enumerated by qPCR of encapsidated genomes. Error bars represent SEM of four independent ORF75.stop1.2 virus stocks and their respective marker rescue viruses generated in parallel. Significance determined by the one-way paired t-test. **(C)** Quantification of the infectious particle to PFU ratio. Infectious particles were enumerated by qPCR of cell-associated viral genomes 2 hpi upon 75A.stop1 or 75A.stop1MR infection of primary MEFs. Ratios of 75A.stop1 viruses are relative to the 75A.stop1MR fraction; significance determined by one-way ANOVA. **(D)** Timecourse analysis of immediate early promoter activity during lytic infection. NIH 3T12 cells were transfected with a RTA responsive immediate-early promoter expressing luciferase, and subsequently infected with equivalent PFU (MOI of 5) or equivalent genomes. **(E)** Timecourse analysis of NF-κB activation during lytic infection. NIH 3T12 cells were transfected with a NF-κB responsive promoter expressing luciferase, and subsequently infected as described in (D). **(F)** Time course analysis of TNFα release in BMDMs infected as described in (D). Dashed line indicates the limit of detection for the assay. Samples that are below the limit of detection (B.L.D.) are indicated. **(G)** Timecourse analysis of LDH release as described in (F). Error bars indicate SD with three independent biological replicates. * p ≤ 0.05 ** p ≤ 0.005, and **** p ≤ 0.00005; significance determined by one-way ANOVA compared to respective 75A.stop1MR at matched timepoints (D-G).

Since we observed more 75A.stop1.2 virions attaching during infection and increased deposition of tegument-derived ORF75C into infected cells, we next investigated if the loss of ORF75A affected the composition of a mature virion. Virions were purified from three independent virus stocks and analyzed via immunoblot. ORF75C is one of the most abundant tegument proteins in the mature virion, while ORF45 is an inner tegument protein in loose association with the viral capsid [5, 6, 37]. Relative to the minor capsid protein ORF65, levels of the ORF75C and ORF45 tegument proteins and the entry determinant glycoprotein B were comparable in 75A.stop and 75A.stopMR virions (Fig 7A). Thus, the absence of ORF75A does not impact several major components of a mature virus particle.

The observation of increased deposition of both ORF75C tegument protein (Fig 5B and 5D) and viral genomes (Fig 5E) suggested that the loss of ORF75A led to the production of a higher ratio of particles to plaque forming units (PFU). To test this, four independent virus stocks were pelleted via ultrcentrifugation on a 20% sucrose cushion followed by a DNase digest to remove any contaminating extracellular viral DNA. Encapsidated DNA was then released and quantified by qPCR as a measurement of particles. Parallel virus stock preparations were titered via plaque assay. The particle to PFU ratio was determined as the number of encapsidated genomes that led to a single plaque. 75A.stop1.2 viruses required eight-fold more virus particles to produce a single plaque compared to 75A.stop1MR virus stocks (Fig 7B).

Our data indicates that 75A.stop1.2 viruses have an increased particle to PFU ratio which leads to an increased number of attached 75A.stop virions when the infection is based on equivalent PFU. We next tested if the increase in encapsidated genomes per PFU of 75A.stop led to the delivery of more virus genomes. First, primary fibroblasts were infected with 75A.stop1 or control viruses, and samples were collected as a cell pellet, conditioned media, or combined as a total fraction. Next, NIH 3T12 fibroblasts were infected with equivalent volumes of each fraction. After virus adsorption for two hours followed by a citric acid wash, the number of viral genomes delivered to cell was determined by quantitative PCR. In a parallel infection with the same volume of the inoculum, the number of infectious particles produced was measured by plaque assay. Thus, the proportion of delivered genomes that led to a single plaque, relative to that of the 75A.stop1MR virus, provided a ratio of infectious particles to PFU (Fig 7C). Consistent with our findings of a higher particle to PFU ratio based on encapsidated genomes (Fig 7B), we observed that 75A.stop1 viruses required nine-fold more infectious particles to initiate plaque formation relative to 75A.stop1MR and the MHV68-H2BYFP control viruses (Fig 7C). We anticipated that virions within the cell might have a greater defect in their infectious particle to PFU ratio. However, 75A.stop1 virus preparations, whether cell-associated or derived from the conditioned media, had a similar nine-fold increase in the amount of particles delivered to the cells for every plaque-forming unit, as compared to 75A.stop1MR virus fractions (Fig 7C). Taken together, infection with equivalent PFU based on standard plaque titration assays on fibroblasts resulted in a nine-fold increased delivery of 75A.stop1 encapsidated viral genomes compared to 75A.stop1MR.

### Enhanced viral gene expression and host responses to 75A.stop1 infection is due to the increased particle delivery

To further examine the consequence of increased tegument deposition upon 75A.stop virus infection, we investigated another well-characterized function of tegument ORF75C. ORF75C has been reported to engage the host PFAS to activate the RIG-I/MAVS pathway, ultimately leading to the activation of upstream signaling kinase of NF-κB, IKKβ [14]. Activation of IKKβ leads to the phosphorylation and transactivation of the major lytic transcription factor, RTA [38]. Due to the increased deposition of ORF75C in cells infected with 75A.stop1.2 at equivalent plaque-forming units per cell (PFU) (Fig 5B and 5D), we hypothesized that 75A.stop1.2 infected cells would have increased RTA promoter activity and NF-κB promoter activity. We infected NIH 3T12 cells with 75A.stop1MR or 75A.stop1.2 at equivalent PFU or input genomes following transfection with an RTA- or an NF-κB-responsive luciferase reporter plasmid. Indeed, there was an increase in immediate-early promoter activity at both 18 hpi and 24 hpi when fibroblasts were infected at an equivalent PFU with 75A.stop1.2 compared to 75A.stop1MR (Fig 7D). However, when fibroblasts were infected with volumes of 75A.stop1.2 viruses required to achieve equivalent input genomes to 75A.stop1MR, the enhanced immediate-early promoter activity was lost, instead it was reduced compared to 75A.stop1MR at both timepoints (Fig 7D). We also observed a transient increase in NF-κB promoter activity at 18 hpi when fibroblasts were infected with 75A.stop1.2 at equivalent PFU (Fig 7E). However, the heightened NF-κB promoter activity was lost when fibroblasts were infected with equivalent genomes of 75Astop1.2 (Fig 7E). Taken together, the increased particle to PFU ratio of 75A.stop virus stocks led to increased immediate-early promoter activity and NK-κB promoter activity at early stages of infection. When the input virus was adjusted to deliver equivalent genomes, a defect in RTA activity and NF-κB induction in the absence of ORF75A was revealed.

We next examined if the increased particle to PFU ratio of the 75A.stop1.2 virions enhanced the pro-inflammatory response in BMDMs. We infected mouse BMDMs with 75A.stop1MR virions or 75A.stop1.2 virions at equivalent PFU or equivalent genomes, and examined the release of TNFα and lactose dehydrogenase (LDH), as indicators for inflammatory signaling and cell death, respectively. Infection of BMDMs infected with 75A.stop1MR viruses produced undetectable levels of TNFα at either timepoint investigated, while TNFα production was observed upon infection with 75A.stop1.2 at equivalent PFU (Fig 7F). However, TNFα production was not detected when BMDMs were infected with equivalent genomes of 75A.stop1.2 (Fig 7F). We next examined LDH release from infected BMDMs. 75A.stop1MR infected BMDMs led to a slight increase in cell death by 24 hpi that increased to approximately twenty percent relative to a positive control by 48 hpi (Fig 7G). In contrast, BMDMs infected with equivalent PFU of 75A.stop1.2 led to a significant increase in cell death at 24 hpi, with accelerated cell death by 48 hpi (Fig 7G). Notably, when BMDMs were infected with 75A.stop1.2 at equivalent genomes there was less cell death compared to the 75A.stop1MR-infected BMDMs (Fig 7G). Taken together, this data indicates that the enhanced particle to PFU ratio observed in virus stocks produced the absence of ORF75A leads to pro-inflammatory responses and cell death. This trend is reversed if the inoculum is adjusted to infect with equivalent genomes of 75A.stop.

### ORF75A is required to drive early events in virus infection in cell culture and acute replication *in vivo*

**Fig 8:**
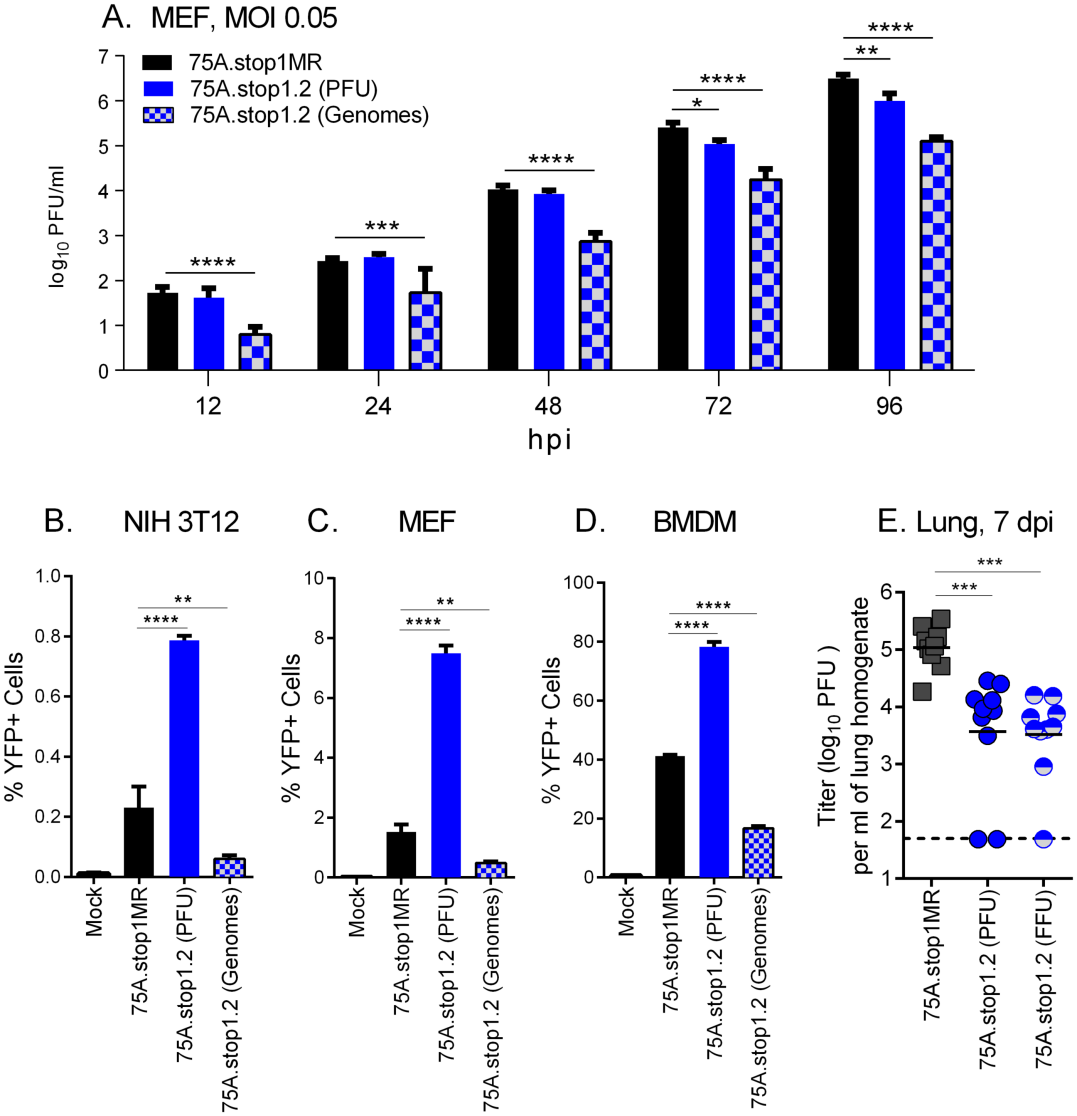
Input of equivalent genomes reveals role for ORF75A in driving productive replication in cell culture and in mice. **(A)** Multi-step growth curve in NIH 3T12 cells infected with equivalent PFU or equivalent genomes. **(B-D)** Expression of the YFP reporter gene in NIH 3T12 fibroblasts **(B)**, primary fibroblasts (MEFs) **(C)**, and primary bone marrow-derived macrophage cells (BMDM) **(D)** upon infection with indicated viruses at an equivalent PFU or with equivalent genomes. **(E)** Acute replication in the lungs of mice 7 dpi. Mice were infected with indicated viruses at 1000 PFU or adjusted to infect at equivalent FFU. * p ≤ 0.05, ** p ≤ 0.005 ***, p ≤ 0.0005, and **** p ≤ 0.00005; significance determined by two-way ANOVA for A and one-way ANOVA for B-E.

The increased particle to PFU led to earlier and increased immediate-early promoter activity (Fig 7). We next investigated if infecting primary fibroblasts with equivalent genomes would ameliorate the detrimental effects on virus production. Primary fibroblasts were infected with equivalent PFU per cell or equivalent input genomes per cell. 75A.stop1.2 infected fibroblasts infected with equivalent PFU fail to reach peak infectious particle production as compared to MR after 48 hpi (Fig 8A). Adjusting the input to deliver equivalent genomes did not improve virus replication; instead this manifested in a further impairment of 75A.stop1.2 replication (Fig 8A).

RTA-responsive promoter activation (Fig 7D) and virus replication (Fig 8A) was markedly reduced in cells infected with equivalent genomes of ORF75Astop. This suggested that there was a defect in launching viral gene expression from the deposited genomes. We next investigated the percentage of cells that express a CMV-IE promoter driven YFP reporter gene from the viruses in multiple cell types. When fibroblasts were infected to achieve an equivalent PFU per cell, we observed an approximate 4-fold increase in the percentage of cells infected with 75A.stop1.2 that were expressing YFP compared to MR-infected cells (Fig 8B–8D). This indicates that the increase in input genomes due to the higher particle to PFU ratio leads to an increase in the percentage of cells infected at a low MOI. Indeed, when fibroblasts were infected based on genome equivalents, cells that express YFP were reduced to significantly lower levels than cells infected with MR (Fig 8B and 8C). In addition, we observed a significant reduction in the percentage of YFP+ BMDMs upon infection with equivalent input genomes of 75A.stop1.2 compared to MR-infected BMDMs (Fig 8D). Taken together, there is an intrinsic defect in gene expression from the input viral genome when ORF75A is absent.

Since many of the replication phenotypes of 75A.stop observed *in vitro* could be attributed to the increased particle to PFU ratio, we wanted to confirm that replication defect in the mice was not attributed to the increased particle to PFU ratio of ORF75A virus stocks. To address this issue, we adjusted the mouse inoculum to deliver either equivalent plaque-forming units or equivalent YFP focus-forming units (FFU), based on NIH 3T12 fibroblast titration (Fig 8B) and analyzed acute replication in the lungs of mice 7 dpi. We observed a similar trend in mice infected with either equivalent PFU or FFU of 75A.stop. Virus replication was significantly reduced in the lungs of mice infected with 75A.stop1.2 compared to MR, regardless of the input inoculum (Fig 8E). Taken together, there is an intrinsic requirement for ORF75A to drive MHV68 replication and expansion in replication in cell culture and the acute phase of infection *in vivo*.

## Discussion

This study is the first to define the role of vFGARAT genes in the context of a latent infection in the host. Upon intranasal inoculation of mice, 75A.stop viruses were impaired in their ability to replicate in the lungs, and exhibited a severe defect in latency establishment and reactivation from latency in the spleen. Bypass of the respiratory tract by intraperitoneal inoculation restored latency establishment, but not reactivation, from latent splenocytes. We observed a nine-fold increase in the particle to PFU ratio of 75A.stop virus stocks, which resulted in an increased deposition of tegument and accelerated viral gene expression in the next round of infection. In addition, the accelerated viral gene expression was discordant with infectious particle production. However, infections that adjusted virus input based on input genomes or equivalent YFP-foci revealed an intrinsic requirement for ORF75A in the early stages of virus infection that led to reductions in replication in cell culture and in mice. ORF75B.stop viruses were not impaired for replication or colonization of latency reservoirs *in vivo*, suggesting that ORF75B has overlapping functions with the other vFGARATs.

We observed two distinct phenotypes *in vivo*, a defect in acute replication in the lungs and a defect in reactivation from splenocytes. These are significant findings since they are two different tissues, lung epithelium and splenocytes, and they reflect two different aspects of the virus lifecycle that involve productive infection, lytic replication in the acute phase and reactivation from latency. The severe defect in latency establishment by intranasal inoculation suggests a role for ORF75A in MHV68 dissemination from initial sites of virus replication to distal latency reservoirs. MHV68 mutants harboring defects in genes necessary for lytic gene expression [39, 40] or DNA replication [41–46] fail to establish WT levels of latency in the spleen in a route-dependent manner. Thus, the acute phase of infection after intranasal inoculation seems to promote the efficient seeding of the latent splenic reservoir. MHV68 replicates in alveolar epithelial cells of the lung [47], and is detected in the MLN prior to, and concomitant, with infection of peripheral blood mononuclear cells [21–24, 48]. These observations support a model whereby the virus gains access to B cells in the lymph nodes that survey sites of acute infection, and then traffics to the spleen via the blood.

Given the route-dependent requirement for ORF75A to establish latency after intranasal inoculation, but not intraperitoneal inoculation, we reasoned that the failure of 75A.stop to expand in the lungs to the same degree as WT virus directly impacted the kinetics of dissemination to the draining MLN, blood, and spleen. We observed that the loss of ORF75A reduced the number of infected cells in the draining MLN and circulating PBMC at 9 dpi, and these levels remained dramatically reduced in the spleen at 16 dpi. Yet, it remains to be determined whether these defects are due to a failure to reach a required threshold of replication in the lungs or might be attributed to effects mediated by the host immune response. Alternatively, these findings might reflect an intrinsic requirement for ORF75A during the establishment of latent infection or reactivation from specific cell types such as myeloid or dendritic cells, in addition to B cells, while in transit from sites of acute infection [49–51].

Bypassing the lung, and the acute replication defect therein, by intraperitoneal inoculation revealed a role for ORF75A in reactivating from the splenocyte population. As further discussed below, in the absence of ORF75A, an increased particle to PFU ratio is required to drive early lytic replication in cell culture, perhaps via increased tegument delivery. The reactivation program in MHV68 infected B cells is not well-characterized, but the contribution of the tegument would not likely occur at the onset. ORF75A transcripts were detected at higher levels in latent B cells stimulated to reactivate by the phorbol ester 12-O-Tetradecanoylphorbol-13-acetate, as compared to transcript levels during de novo infection [18]. Thus, it is interesting to hypothesize that ORF75A has a role in initiating early viral transcription in this context. The disruption of ATRX by the Daxx interaction domain of EBV BNRF1 is critical for the expression of viral genes required to establish latency programs that drive B cell immortalization, likely via the prevention of chromatin modifications that silence newly delivered viral genomes [52–55]. Perhaps ORF75A functions in a similar manner to induce early lytic replication. However, there is no reactivation defect in the PECs in the absence of ORF75A, perhaps indicating differences in epigenetic modifications in different cell types.

The *in vivo* observations led us to more closely examine ORF75A in cell culture systems permissive for lytic replication. In both primary MEFs and BMDMs, loss of ORF75A reduced the production of infectious virus. This was coupled with an unexpected increase in intracellular viral protein expression across all kinetic classes, increased viral mRNA levels, and increased deposition of tegument-derived ORF75C in subsequent rounds of infection. We investigated the particle to PFU ratio of the 75A.stop1.2 virions and observed a nine-fold increase in the amount of particles required to form a plaque, which led to the increased deposition of tegument and viral genomes in the next round of 75A.stop infection.

The increase in the particle to PFU ratio of 75A.stop led to enhanced NF-κB activity and activation of immediate-early promoters. This phenomenon might be attributed to either an increase in the deposition of viral genomes or to an increased deposition of one or more tegument proteins. For instance, the virion-associated protein ORF49 promotes MHV68 replication in culture and in mice, and displaces the co-repressor PARP-1 from RTA [56]. ORF75C is another candidate tegument protein since it activates the RIG-I/MAVS pathway [14, 57] and drives the degradation of PML, a factor that dampens MHV68 replication in cells culture and reactivation in vivo [13, 15, 36]. We examined whether increased ORF75C tegument deposition correlated with accelerated PML degradation in 75A.stop infected cells. However, rates of PML degradation did not differ between 75A.stop1 and 75A.stop1MR infections. In addition, we generated a mutant lacking a putative E3 ligase domain of ORF75C required for PML degradation [13] in combination with the 75A.stop mutation. The loss of PML degradation by ORF75C did not reverse the enhancement in lytic gene expression or the decrease in infectious virus production that characterized the 75A.stop mutant. Thus, the mechanism of accelerated viral gene expression triggered by the loss of ORF75A is independent of ORF75C-driven PML degradation.

Interestingly, two vFGARATs, MHV68 ORF75C and KSHV ORF75, have been reported to interact with the human FGARAT (PFAS) to direct RIG-I deamidation and engagement of MAVS to activate IKK2 [14]. IKK2 activation promotes the transactivation of lytic genes by the viral protein RTA [14, 38]. We observed that the enhanced immediate-early promoter activity was dependent on an increased particle to PFU ratio of the 75A.stop1.2 virus. Thus, the increased delivery of ORF75C might heighten RTA activity and accelerate viral gene expression.

MHV68 mutants lacking mLANA exhibit dysregulation of gene expression coupled to a reduction in infectious particle output, and this is coupled to an increase in cell death [58]. We report here that higher 75A.stop particle input led to an increase in TNFα production and cell death in BMDMs. The MHV68 muSOX mutant lacking the host shutoff function yields virions with an altered tegument and glycoprotein composition that leads to enhanced binding and entry into target cells, but decreased viral progeny [59]. ORF75A has been reported in virions upon immunoprecipitation [1], but not by mass spectrometry of virions [5, 6, 59], indicating ORF75A is not a major component of MHV68 virions. We did not detect any significant changes in ORF75C or ORF45, two well-defined tegument proteins, in the virions produced in the absence of ORF75A, suggesting there are no major alterations in virion composition. Further investigations to identify any slight changes in virion composition would require quantitative mass proteomics approaches.

Recognizing the impact of the higher particle delivery, we adjusted our input such that equivalent genomes were delivered and found that infections with 75A.stop led to a lower percentage of IE gene expression and YFP-expressing cells. This indicates that ORF75A is needed in the earliest stages of *de novo* infection. The defined mechanisms by which the vFGARATs target and inhibit antiviral components of PML-NBs [3, 4, 10, 11, 15, 60] are strikingly divergent. During MHV68 infection, the only PML-NB component that appears to be targeted by degradation is PML, via an E3 ligase function of MHV68 ORF75C [10, 15]. While ORF75A does not impact the levels of PML-NB components examined to date [4, 10], its impact on relocalization of those components is not well-characterized. Future studies will investigate the mechanism by which ORF75A promotes early viral events in lytic replication, as well as any contribution of ORF75A in disarming PML-NBs.

In summary, we have identified a novel role for a vFGARAT protein in MHV68 replication and pathogenesis. Virus particles produced in the absence of ORF75A are impaired for replication in cell culture, at a site of acute infection *in vivo*, and are less efficient in their ability to reactivate from latency in the spleens of infected mice. An alteration in the ratio of particles to PFU of a herpesvirus mutant can have unexpected consequences for virus replication. In cell culture studies, the infection of 75A.stop mutants based on equivalent PFU led to an acceleration in viral gene expression in fibroblast cells and enhanced TNFα release and increased cytotoxicity in infected BMDMs. Upon adjusting virus infections to deliver equivalent genomes and thereby lower input PFU, these phenotypes were reversed, and the 75A.stop virus was found to be defective in driving IE gene expression. The determination of PFU by plaque titration assays requires one to enumerate well-isolated plaques. Thus, plaque assays are effectively low MOI and reveal failures of single particles to effectively drive replication forward through multiple rounds of replication to produce a clearing in the monolayer. This defect in plaque formation is consistent with the replication defect in the lungs of infected mice and upon splenic reactivation, other restrictive and likely low MOI infection events. Importantly, the consequences of ORF75A loss are quite distinct from those of ORF75B and ORF75C and this analysis of the ORF75A vFGARAT suggests that there are additional roles for the vFGARATs, distinct from the previously reported functions to antagonize PML-NBs.

### Ethics statement

All protocols were carried out under adherence with the Guide for the Care and Use of Laboratory Animals of the National Institutes of Health and the American Veterinary Medical Association Guidelines on Euthanasia. The animal protocol was approved by the Institutional Animal Care and Use Committee of Stony Brook University (approval number 253637).

## Materials and methods

### Mice and cells

WT C57BL/6 mice were purchased from Jackson laboratories (Bar Harbor, Maine) or bred at the Stony Brook University Division of Laboratory Animal Research (DLAR) facility. All protocols were approved by the Institutional Animal Care and Use Committee of Stony Brook University. Primary murine embryonic fibroblast (MEF) cells were isolated from C57BL/6 mice and maintained in Dulbecco’s modified Eagle’s medium supplemented with 10% fetal calf serum, 100 U of penicillin per ml and 100 mg of streptomycin per ml at 37°C in 5% CO_2_. To generate bone marrow-derived macrophages (BMDMs), bone marrow from 8–12 week old mice was flushed from the femur and differentiated for 5 days in DMEM with Glutamax (Life Technologies, Grand Island, NY) containing 10% FBS and 30% L-supplement in non-tissue culture treated plates. Cells were maintained in DMEM with Glutamax containing 10% FBS and 10% L-supplement in non-tissue culture treated plates. Immortalized murine fibroblast cells (NIH 3T3 or NIH 3T12) were maintained in Dulbecco’s modified Eagle’s medium (DMEM) supplemented with 8% fetal calf serum, 1% L-glutamine, 100 U of penicillin per ml, and 100 mg of streptomycin per ml at 37°C in 5% CO_2_.

### Phylogenetic analysis and vFGARAT alignment

Phylogenetic analysis and pairwise alignment were performed in Geneious 10.1.2 [61]. The phylogenetic tree was constructed using global alignment with free end gaps and BLOSUM62 cost matrix. Genetic distances were determined by Jukes-Cantor model, and the phylogenetic tree was built with the Neighbor-Joining method. Multiple pair-wise alignments to determine percent identity and percent similarity were built using global alignment and a BLOSUM62 cost matrix.

### Generation of recombinant viruses

The modified MHV68-H2BYFP genome cloned into a BAC was a kind gift from the Speck laboratory [62]. Viruses with stop mutations in *ORF75A* (75A.stop1, 75A.stop2 and ORF75A.dblstop) and *ORF75B* (ORF75B.stop1 and ORF75B.dblstop), in addition to a deletion of aa 648–659 of ORF75C [13] were generated on either wild-type or 75A.stop1 MHV68-H2BYFP BAC by *en passant* mutagenesis [63]. Additionally, the 3XFLAG-ORF75A tagged virus was generated on the MHV68-H2BYFP BAC by *en passant* mutagenesis.

Briefly, forward and reverse primers (S2 Table) containing each ORF75 mutation (underlined), flanking WT ORF75 sequences on either side of the mutation and sequences complementary to the kanamycin selection marker were used to amplify the kanamycin selection marker from plasmid pEPKanS2 [63] by PCR (One Taq DNA polymerase, New England Biolabs, Ipswich MA). This PCR product was excised from the gel, digested with *Dpn*I to remove input template, and transformed into freshly prepared electrocompetent *E*. *coli* harboring the MHV68-H2BYFP BAC. After recovery, the bacterial cells were plated on dual chloramphenicol (34 μg/ml) and kanamycin (50 μg/ml) plates and incubated at 30°C for 48 hrs. DNA was prepared from isolated colonies and the kanamycin selection marker in *Orf75A* or *Orf75B* was PCR amplified to verify the insertion of the mutagenesis cassette into the MHV68-H2BYFP BAC. Next, the kanamycin selection marker was removed, leaving behind the desired ORF75A, ORF75B or ORF75C mutation. The *Orf75A* or *Orf75B* gene was PCR amplified from the putative mutant BAC, digested with a restriction enzyme unique to each mutation to confirm the presence of each stop codon. Insertion of a proximal stop codon into *ORF75A* resulted in a new Mlyl site whilst the more distal stop codon resulted in the gain of a unique SpeI site. In *ORF75B*, the insertion of a proximal stop codon resulted in a loss of an AseI whilst the more distal stop codon resulted in the loss of PstI. Lastly, the *Orf75A* and *Orf75B* genes were PCR amplified and submitted to Laragen (Culver City, CA, USA) for sequencing. To generate marker rescue viruses, primers flanking WT ORF75A or ORF75B sequence were used to generate a targeting construct, that was then used to repair the 75A.stop or ORF75B.stop disrupted viruses described above. Virus passage and titer determination were performed as previously described [64].

### Analysis of recombinant viral BAC DNA

BAC DNA was prepared by Qiagen column purification. For restriction analysis,10 μg of BAC DNA was digested overnight with the desired enzyme and then resolved in a 0.8% agarose gel in 1X TAE. For complete genome sequencing of 75A.stop and ORF75B.stop viruses, the BAC DNA samples were prepared for multiplex, 50-cycle, single-end read sequencing on an Illumina HiSeq2000 by the SUNY-Buffalo Next Gen Sequencing Core. Reads were demultiplexed using the CASAVA 1.8.2 utility program and variants were analyzed as previously described [45]. For complete genome sequencing of the Flag-75A virus, 1 μg BAC DNA was prepared for multiplex, 200-cycle, paired-end read sequencing on an Illumina MiSeq by the Stony Brook Microarray Facility and analyzed as previously described [65]. For alignment of 75C Δ648–659 virus mutant, paired end DNA-seq reads were aligned against the reference using bowtie2 [66] with default parameters. Variants were detected using the FreeBayes tool [67] at depth coverage of at least 500 where the minimum base quality and mapping quality requirements were set to 20 on the Phred quality scale.

### Virus infections and growth curves

1.2 x 10^5^ fibroblasts were seeded into each well of a 12-well tissue culture plate one day prior to infection with recombinant MHV68. To determine tegument-delivered ORF75C, NIH 3T12 cells were treated with or without cycloheximide (200 ng/ μL) beginning one hour prior to infection. To measure virus replication, fibroblasts or bone-marrow derived macrophages were infected at indicated MOIs. Triplicate wells were harvested for each timepoint, and the cells with the conditioned medium were stored at -80°C. Serial dilutions of cell homogenate were used to infect NIH 3T12 cells and then overlaid with 1.5% methylcellulose in DMEM supplemented with 5% FBS. For virus growth in bone-marrow derived macrophages, 2 x 10^5^ cells were seeded into each well of a 12-well tissue culture plate two days prior to infection at an MOI of 5. Triplicate wells were harvested for each timepoint, and the cell homogenate was stored at -80°C. One week later, the methylcellulose was removed and cells were washed twice with PBS prior to methanol fixation and staining with a 0.1% crystal violet solution in 10% methanol to allow visualization of plaques.

### Infections and organ harvests

8 to 10 week old WT mice were infected with 1000 PFU of MHV68 either by intranasal inoculation in a 20 μl bolus or by intraperitoneal injection of 0.5 ml with 1000 PFU of MHV68 under isoflurane anesthesia. The inoculum was back-titered to confirm the infectious dose. Mice were sacrificed by terminal isoflurane anesthesia. For acute titers, mouse lungs were harvested in 1 ml of DMEM supplemented with 10% FBS and stored at -80°C prior to disruption in a Mini-BeadBeater (BioSpec, Bartlesville, OK). The homogenates were titered by plaque assay. For latency and reactivation experiments, mouse spleens were homogenized, treated to remove red blood cells, and then filtered through a 100 μm nylon filter. For peritoneal cells, 10 ml of media was injected into the peritoneal cavity and an 18-gauge needle was used to withdraw approximately 7 ml of media from each mouse. The peritoneal exudate cells were pelleted by centrifugation and then resuspended in 1 ml of DMEM supplemented with 10% FBS.

### Limiting dilution analysis of latency and reactivation

To determine the frequency of cells harboring the viral genome, single cell suspensions were prepared for single-copy sensitivity nested PCR, as previously described [68, 69]. To determine the frequency of cells harboring latent virus capable of reactivation upon explant, single cell suspensions were prepared from mice 16 or 18 dpi, resuspended in DMEM supplemented with 10% FBS and plated in twelve serial two-fold dilutions onto a monolayer of MEFs prepared from C57BL/6 mice in 96-well tissue culture plates, as previously described [45].

### Antibodies and immunoblotting

Total protein lysate was harvested in lysis buffer (150 mM sodium chloride, 1.0% IGEPAL CA-630, 0.5% sodium deoxycholate, 0.1% sodium dodecyl sulfate, 50 mM Tris [pH 8.0]) supplemented with a protease inhibitor cocktail (Sigma, St. Louis, MO) and phenylmethylsulfonyl fluoride. Portions of each lysate were separated on a 10% SDS-PAGE gel or on a gradient 4 to 15% SDS-PAGE gel (Bio-Rad, Hercules, CA) and transferred to polyvinylidene fluoride membrane.

Commercially available monoclonal antibodies were used to detect FLAG expression with FLAG-M2 (Sigma) and mouse PML (EMD Millipore). Antibodies used to detect other lytic proteins include: affinity-purified chicken anti-peptide antibodies to ORF59 [70] and ORF75C [45] (Gallus Immunotech, Fergus, Ontario, Canada), rabbit polyclonal antibodies to ORF45 [71] and ORF65 (M9) [72] (kindly provided by Dr. Ren Sun, University of California at Los Angeles), ORF50 (RTA) [38] (kindly provided by Dr. Pinghui Feng, UT Southwestern Medical Center), ORF73 (LANA) [73] (kindly provided by Dr. Scott Tibbetts, University of Florida), and ORF57 [15] (kindly provided by Dr. Paul Ling, Baylor College of Medicine), and mouse polyclonal glycoprotein B [74] (kindly provided by Dr. Philip Stevenson, University of Cambridge). A rabbit polyclonal antibody to GAPDH (Sigma) was used as a loading control. Horseradish peroxidase-conjugated secondary antibodies were detected using an enhanced chemiluminescence reagent (ECL; Thermo Scientific). Chemiluminescent signals (ThermoScientific, Boston, MA, USA) were detected by audioradiography film or a LAS 500 Chemiluminesence Imager (GE Healthcare).

### Northern blotting

The northern blot protocol for longer RNA molecules (>200) was as follows: 3.5 μg total RNA from MHV68-infected or mock-infected murine fibroblasts was loaded onto 1% agarose gel containing 6% formaldehyde in parallel with RNA Millenium Marker (Ambion). The gel was run in MOPS buffer, then blotted onto an Hybond XL nylon membrane (Life Technologies) overnight with Turblotter kit in 20X SSC buffer. Following washing, the RNA was crosslinked to the membrane by UV, and the membrane was stained with 0.02% methylene blue for visualization of RNA integrity and markers. The membrane was then pre-hybridized at 63°C for 4 hours in ULTRAhyb (Ambion) buffer. The ^32^P-labeled RNA probes were generated with Maxiscript T7/Sp6 Transcription Kit (Ambion) using PCR amplicons specific for ORF75A/B/C regions (S3 Table). Subsequently, RNA probes were added and incubated overnight. Following washes with 1x SSC buffer, the membrane was exposed to film at 80°C.

### Quantitative PCR

Total cell DNA from infected MEFs was isolated using the DNeasy Blood and Tissue kit (Qiagen; Limburg, Netherlands). A total of 25 ng of template DNA was used in the quantitative PCR (SYBR green low ROX mix; Thermo Scientific, Waltham, MA) using primers specific to a region of MHV68 ORF50 as previously described [45].

### Virion purification

NIH 3T12 cells were inoculated with virus at an MOI of 0.05. Infected cells were harvested when the monolayer exhibited ~80% cytopathic effect. Infected fibroblasts and the conditioned media was collected and dounced to release intact virus particles. Subsequently, cellular debris was pelleted at low centrifugation 5,000×g. The virus was then concentrated via high speed centrifugation (20,000×g) in a Sorvall Superspeed Centrifuge (Du Pont Instruments). Concentrated virus pellet was then resuspended and pelleted in a Beckman L7-65 Ultracentrifuge (~60,000×g) on a 20% sucrose cushion. The virus pellet was then concentrated and banded through a sucrose gradient (10%, 20%, 30%, 40%, 50%, 60%) at ~70,000×g. The purified virus band was isolated, pelleted via ultracentrifugation, and lysed for immunoblot analysis.

### Quantification of the particle to PFU ratio

Virions from independent virus stocks were pelleted via ultracentrifugation on a 20% sucrose cushion. These pelleted virions were resuspended and treated with TURBO DNA-free kit to remove any contaminating extracellular viral DNA following the manufacture’s protocol (Invitrogen). Samples were subsequently incubated at 72°C to release the encapsidated viral DNA. Released viral DNA was isolated via High Pure Viral Nucleic Acid Kit (Roche) manufacturer’s protocols. Purified viral DNA samples were enumerated via qPCR and divided by the PFU of the viral stock to determine the particle to PFU ratio. To confirm the samples were sufficiently digested we analyzed GAPDH levels via qPCR. After DNase digestion, GAPDH levels were reduced to nearly undetectable levels.

### Determination of infectious particle to PFU

MEFs were infected at a MOI of 0.05 in triplicate and harvested three days later. Samples were isolated as a cell pellet, conditioned media, or combined as a total fraction. All samples were cryogenically disrupted three times. NIH 3T12 cells were used to quantify delivered genomes and infectious particles. After virus adsorption, infected cells were washed with PBS, followed by two citric acid washes (40 mM Na_3_C_6_H_5_O_7_, 10 mM KCL, 135 mM NaCl, pH 3.0). To determine the quantity of infectious particles, viral genomes were quantified 2 hpi by quantitative PCR. Equivalent infection volumes were used in parallel for titration of plaque-forming units by standard plaque assay. After infection, DMEM supplemented with 8% FBS was added to the wells. The infectious particle to PFU was generated by dividing the normalized genomes (ORF50/GAPDH) delivered 2 hpi by the quantity of infectious particles (PFU). The infectious particle to PFU was calculated for each sample relative to the infectious particle to PFU of the control 75A.stop1MR.

### Luciferase assay

To examine ORF57 or NF-κB promoter transactivation in the context of infection, NIH 3T12 cells were seeded at 8.0 × 10^5^ cells per 10 cm dish one day prior to transfection with 10 μg of pORF57-luc or pGL-4.32 by TranIT-X2 (Mirus). The next day, 1.2 × 10^5^ cells per well were seeded into 12-well plates and then infected the following day with 75A.stop1.2 or revertant viruses based on equivalent PFU or input genome equivalents per cell. Samples were harvested at indicated timepoints, and lysed in 1X passive lysis buffer (Promega, Madison, WI). Luciferase assays were normalized to total protein by Nanodrop A_280_.

### ELISA and cell death assay

TNFα was quantified by using mouse Duoset TNFα ELISA kit (R&D Systems, Minneapolis, MN). Plates were prepared and assayed according to the manufacturer’s protocol. The samples absorbance was analyzed at 450 nm. Lactate dehydrogenase (LDH) release into the conditioned medium was measured using a CytoTox 96 nonradioactive cytotoxicity assay according to the manufacturer’s instructions (Promega, Madison, WI).

### Statistical analyses

Data were analyzed using GraphPad Prism Software (Prism 5, La Jolla CA). Statistical significance was determined using an ANOVA test followed by Bonferroni correction, paired and unpaired two-tailed or one-tailed t-tests. Under Poisson distribution analysis, the frequencies of latency establishment and reactivation from latency were determined by the intersection of nonlinear regression curves with the line at 63.2.

## Supporting information

S1 Fig ORF75B.stop virus displays no latency or reactivation defect in mice. **(A)** Frequency of splenocytes harboring genomes **18** dpi. ORF75B mutants are red and WT control viruses are black. **(B)** Frequency of splenocytes spontaneously reactivating from latency 18 dpi. For the limiting dilution analyses, curve fit lines were determined by nonlinear regression analysis. Using Poisson analysis, the intersection of the nonlinear regression curves with the dashed line at 63.2% was used to determine the frequency of cells that were either positive for the viral genome or reactivating virus. Data is generated from 2 independent experiments with 4 mice per group. Error bars indicate SEM. * p ≤ 0.05, *** p ≤ 0.0005, and **** p ≤ 0.00005.
(TIF)

S2 Fig Loss of ORF75A does not impair chronic latency. C57BL/6 mice were infected at 1000 PFU by the intraperitoneal route with the indicated viruses. Frequency of splenocytes harboring genomes at six weeks post-infection. For the limiting dilution analyses, curve fit lines were determined by nonlinear regression analysis. Using Poisson analysis, the intersection of the nonlinear regression curves with the dashed line at 63.2% was used to determine the frequency of cells that were either positive for the viral genome or reactivating virus. Error bars indicate SEM. Data is generated from 2 independent experiments of 5 mice per group at 46–60 dpi.
(TIF)

S3 Fig Characterization of ORF75A protein expression. **(A)** Schematic of Flag-75A recombinant virus. **(B)** Single-step growth curve of 75A.stop mutants and WT viruses in the immortalized murine fibroblast line, NIH 3T12 (MOI 5). Error bars indicate SD. **(C)** Timecourse analysis of ORF75A expression with immediate-early (ORF57) and late (ORF65 and ORF75C) gene products upon a single-step infection (MOI 5). **(D)** Immunofluorescence of NIH 3T3 cells transfected with a FLAG-ORF75A expression construct, followed by 24 h infection with MHV68-H2BYFP (MOI of 5). **(E)** Quantification of ORF75A cellular localization. Two individuals independently scored at least 100 cells of each sample, for two independent sample sets. *** p ≤ 0.0005.
(TIF)

S4 Fig Accelerated gene expression coupled with replication defect upon high MOI infection in MEFs. **(A)** Single-step growth curve in MEFs at an MOI of 5 with 75A.stop1.2 and 75A.stop1MR. **(B)** Timecourse analysis of gene products upon a single-step infection of MEFs.
(TIF)

S5 Fig Longer exposure with ORF75C probe reveals the *orf*75A/B/C transcript. Northern blot analysis of NIH 3T12 fibroblast cells infected with indicated viruses at an MOI of 5. Membrane was hybridized with same strand-specific ^32^P-labled cDNA of ORF75C or 18S as described in Fig 5F. ORF75C blot was exposed for 24 hours.
(TIF)

S6 Fig Increased tegument delivery of ORF75C in BMDMs. Immunoblot analysis of ORF75C tegument protein levels 3 hpi of primary BMDMs (MOI 5.0).
(TIF)

S1 Table GenBank accession numbers of vFGARATs. (DOCX)

S2 Table Mutagenic primers used in the generation of the ORF75.stop viruses. (DOCX)

S3 Table Northern blot probes. (DOCX)
