# Correction: Viral FGARAT ORF75A promotes early events in lytic infection and gammaherpesvirus pathogenesis in mice

**DOI:** 10.1371/journal.ppat.1007319

**Published:** 2018-09-25

**Authors:** Nick D. Van Skike, Nana K. Minkah, Chad H. Hogan, Gary Wu, Peter T. Benziger, Darby G. Oldenburg, Mehmet Kara, Deborah M. Kim-Holzapfel, Douglas W. White, Scott A. Tibbetts, Jarrod B. French, Laurie T. Krug

There are several errors in the article.

In the Introduction, there is an error in the second sentence of the third paragraph. The correct sentence is: MHV68 ORF75C and KSHV ORF75 lead to the non-canonical deamidation of RIG-I to drive NF-κB signaling. In the case of ORF75C, this is through the recruitment of the host FGARAT to RIG-I [14].

In the Results section, under the heading “Evolutionary divergence within the vFGARATs,” there is an error in the third sentence of the second paragraph. The correct sentence is: "The C-terminal portion of ORF75C is sufficient to interact with RIG-I [14].

In the Discussion section, there is an error in the first sentence of the seventh paragraph. The correct sentence is: "Interestingly, MHV68 ORF75C has been reported to direct RIG-I deamidation by host FGARAT, and thereby drive MAVS/IKK2/NF-κB signaling [14].

Fig 1B contains a label error. Please find the corrected Fig 1 here.

**Fig 1:**
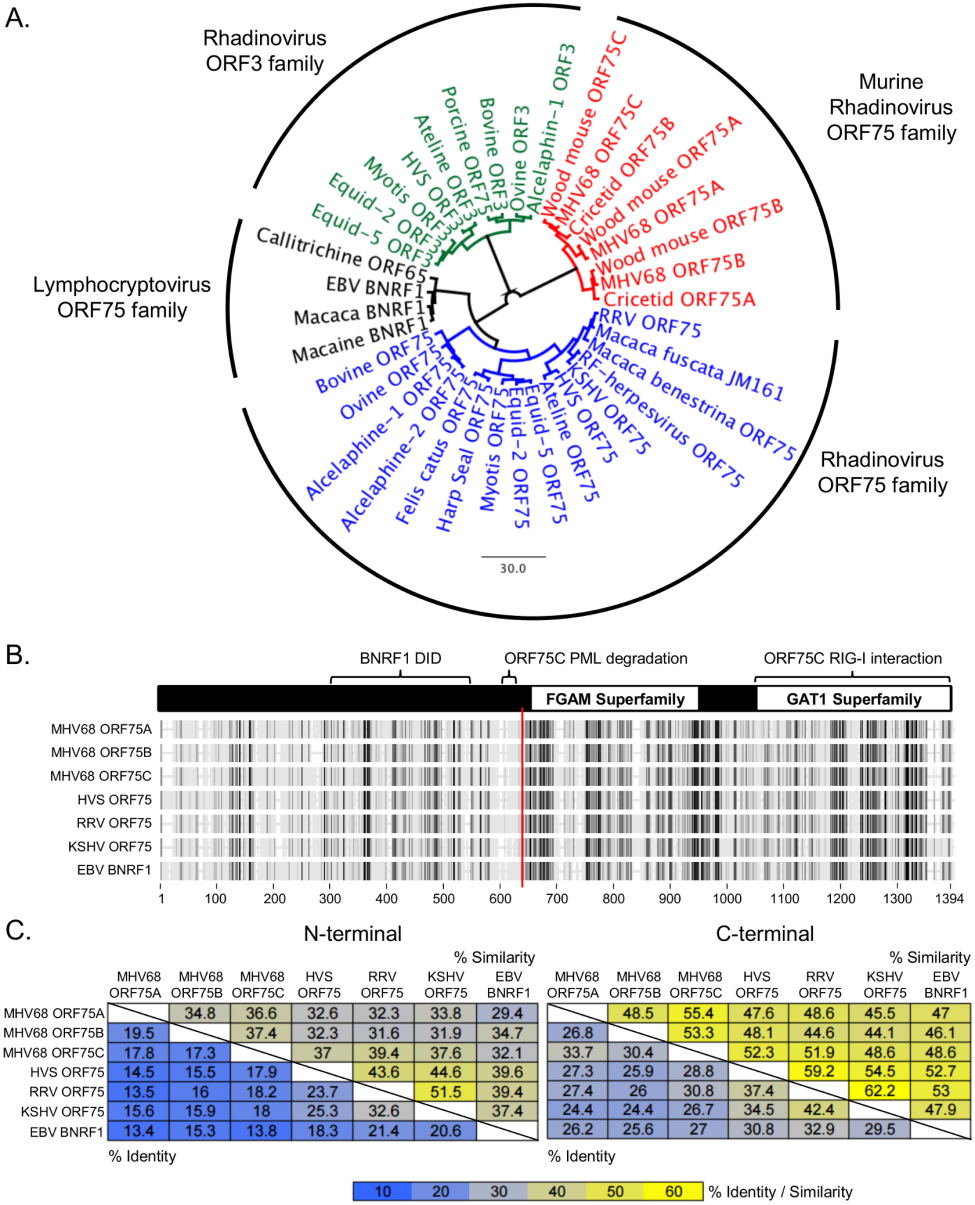
Divergence of the gammaherpesvirus vFGARAT family. **(A)** Phylogenetic tree of the vFGARATs of the indicated γ-hepesviruses (Genebank ID located in S1 Table). **(B)** Phylogenetic alignment using BLOSUM62 with previously characterized domains of the vFGARATs. **(C)** Pairwise alignment showing degrees of identity (lower left corner of table) or similarity (upper right corner of table) between the respective N-terminal or C-terminal portions of the indicated vFGARATs. The division point for each vFGARAT (red vertical line in B) was based on dotplot analysis for each indicated vFGARATS compared to MHV68 ORF75C.

In Table 2, there is an error in the header of the sixth column. Please find the correct Table 2 here.

**Table 2:** Frequencies of cell populations reactivating viral genomes in C57BL/6 mice.

Virus^a^	Route of infection^b^	Organ^c^	dpi	Total # of cells harvested	Frequency of reactivating splenocytes (one in x cells)^d^	Total # of cells reactivating latent virus ^e^
**75A.stop1MR**	i.n.	Spleen	16	9.1 x10^8^	4.1 x 10^3^	2.2 x 10^5^
**75A.stop1.2**	i.n.	Spleen	16	3.6 x10^8^	5.2 x 10^5^	6.9 x 10^2^
**75A.stop2**	i.n.	Spleen	16	0.6 x10^9^	6.9 x 10^5^	8.7 x 10^2^
**75A.dbl.stop**	i.n.	Spleen	16	0.7 x10^9^	6.7 x 10^5^	1.0 x 10^3^
**75A.stop1MR**	i.p.	Spleen	18	5.0 x10^8^	2.0 x10^4^	2.5 x 10^4^
**75A.stop1.2**	i.p.	Spleen	18	3.6 x10^8^	1.0 x10^5^	3.6 x 10^3^
**75A.stop1MR**	i.p.	PEC	18	4.4 x10^7^	3.0x10^3^	1.5 x 10^4^
**75A.stop1.2**	i.p.	PEC	18	3.5 x10^7^	2.1x10^3^	1.6 x 10^4^
**75B.stop1**	i.n.	Spleen	16	1.3 x10^9^	6.1x10^3^	2.1 x 10^5^
**75B.dbl.stop**	i.n.	Spleen	16	0.9 x10^9^	6.8x10^3^	1.3 x 10^5^
**75B.stop1MR**	i.n.	Spleen	16	1.5 x10^9^	6.8x10^3^	2.2 x 10^5^

^a^ Infection with recombinant MHV68 viruses

^b^ i.n., intranasal; i.p., intraperitoneal

^c^ Organ harvested for limiting dilution analysis (MLN, mediastinal lymph node PBMC, peripheral blood mononuclear cell)

^d^ The frequency data were determined from the mean of two to five independent experiments with cells from the indicated organs. Organs were pooled from three to five mice per experiment.

^e^ The total number of cells reactivating latent virus per mouse was extrapolated using the frequency value generated from the limiting dilution analysis together with the total number of splenocytes or PEC cells harvested.
