# Supplementary figures and images for: Viral FGARAT ORF75A promotes early events in lytic infection and gammaherpesvirus pathogenesis in mice
Source: PLoS Pathog. 2018 Feb 1;14(2):e1006843. doi: 10.1371/journal.ppat.1006843 (PMC5811070; doi:10.1371/journal.ppat.1006843)

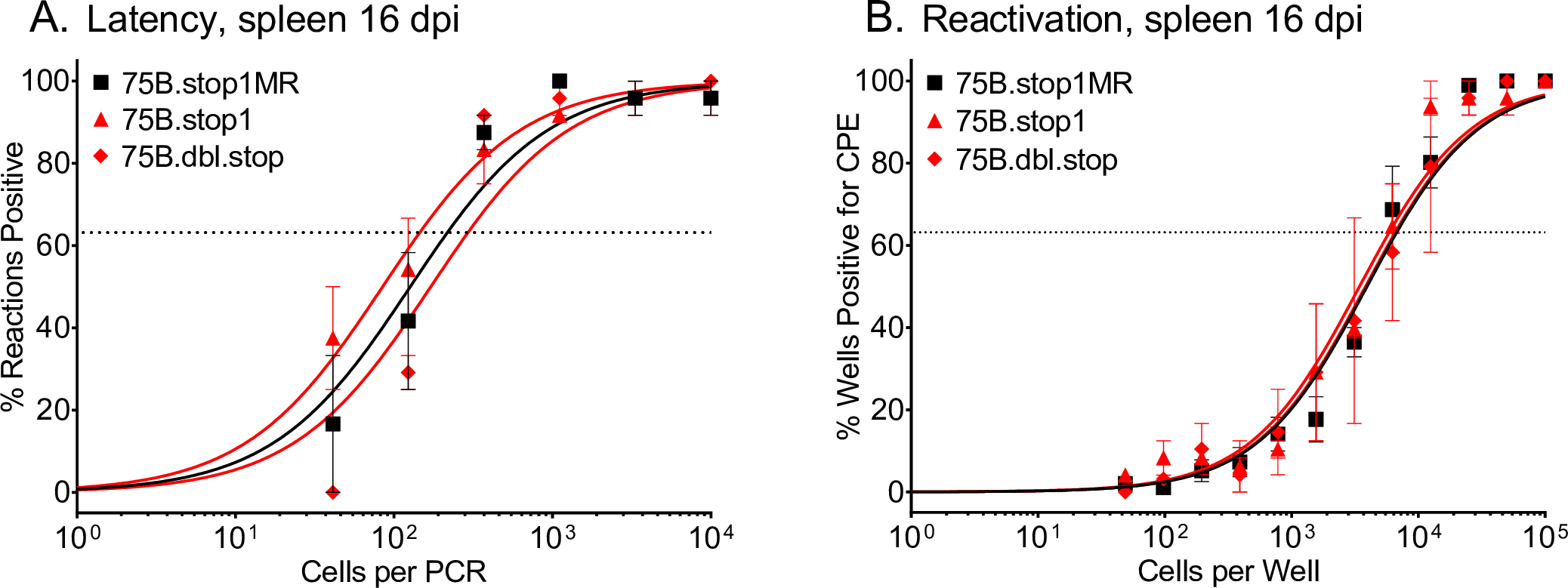

Supplement: S1 Fig — (A) Frequency of splenocytes harboring genomes 18 dpi. ORF75B mutants are red and WT control viruses are black. (B) Frequency of splenocytes spontaneously reactivating from latency 18 dpi. For the limiting dilution analyses, curve fit lines were determined by nonlinear regression analysis. Using Poisson analysis, the intersection of the nonlinear regression curves with the dashed line at 63.2% was used to determine the frequency of cells that were either positive for the viral genome or reactivating virus. Data is generated from 2 independent experiments with 4 mice per group. Error bars indicate SEM. * p ≤ 0.05, *** p ≤ 0.0005, and **** p ≤ 0.00005. (TIF) [file ppat.1006843.s001.tif]

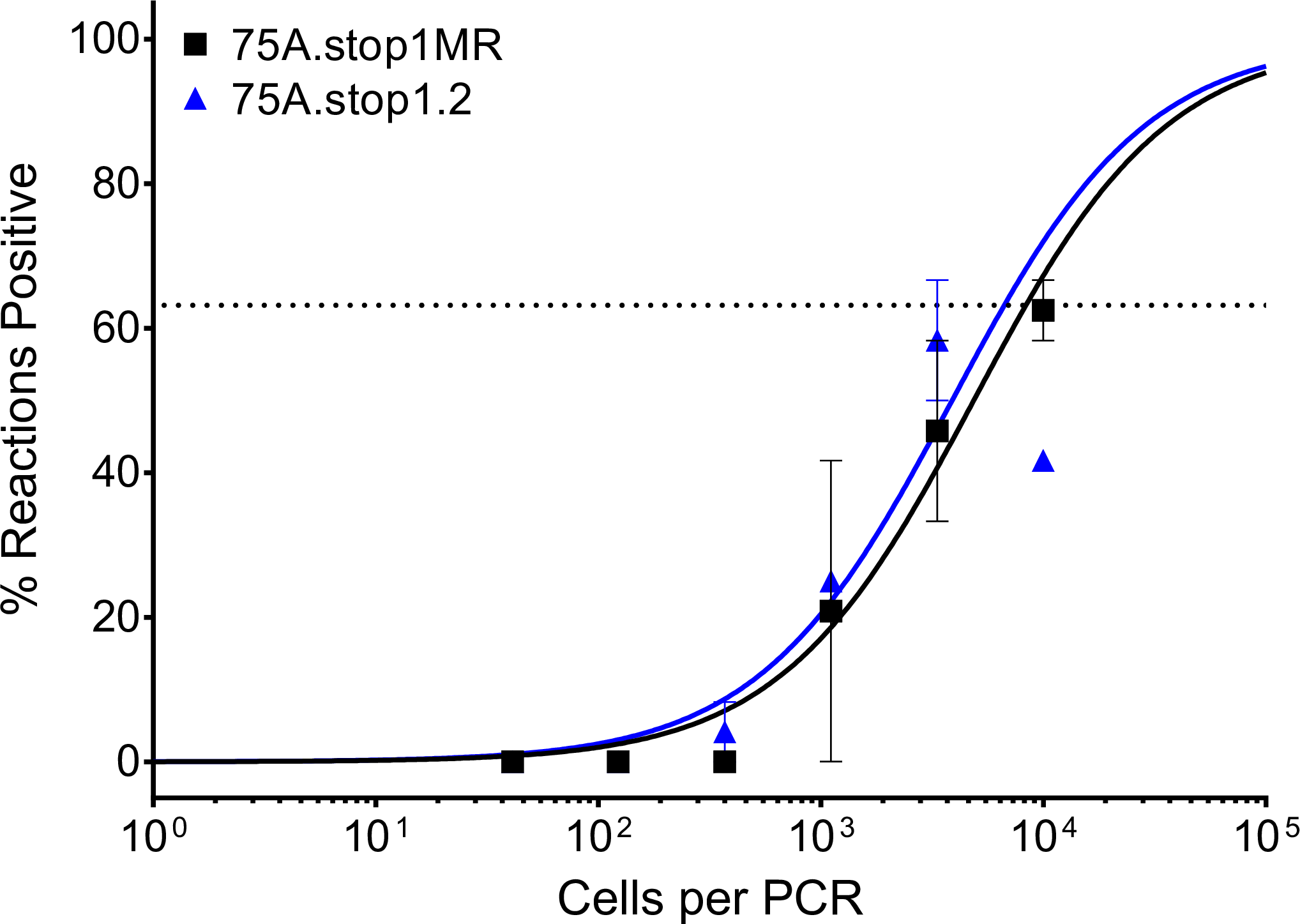

Supplement: S2 Fig — C57BL/6 mice were infected at 1000 PFU by the intraperitoneal route with the indicated viruses. Frequency of splenocytes harboring genomes at six weeks post-infection. For the limiting dilution analyses, curve fit lines were determined by nonlinear regression analysis. Using Poisson analysis, the intersection of the nonlinear regression curves with the dashed line at 63.2% was used to determine the frequency of cells that were either positive for the viral genome or reactivating virus. Error bars indicate SEM. Data is generated from 2 independent experiments of 5 mice per group at 46–60 dpi. (TIF) [file ppat.1006843.s002.tif]

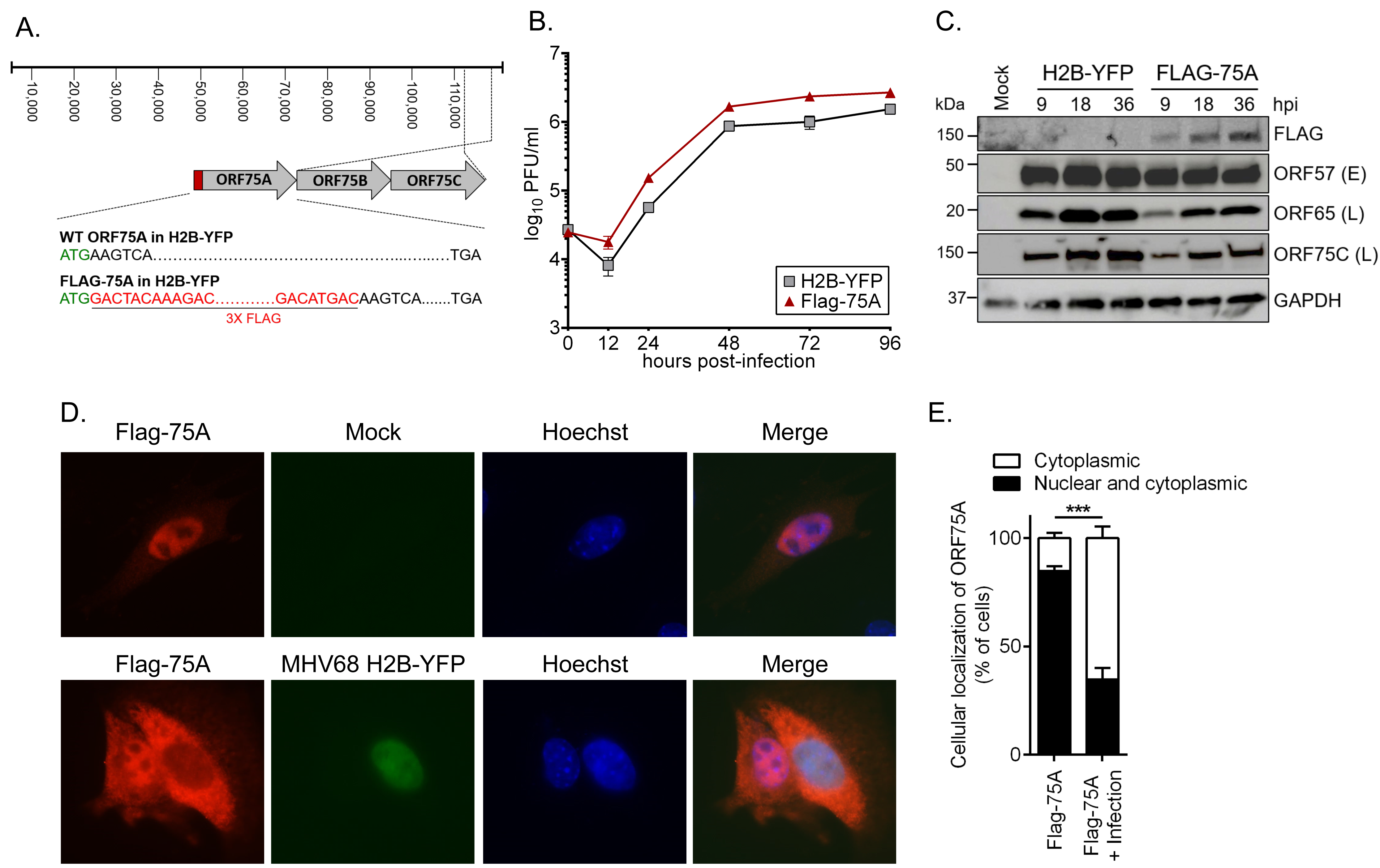

Supplement: S3 Fig — (A) Schematic of Flag-75A recombinant virus. (B) Single-step growth curve of 75A.stop mutants and WT viruses in the immortalized murine fibroblast line, NIH 3T12 (MOI 5). Error bars indicate SD. (C) Timecourse analysis of ORF75A expression with immediate-early (ORF57) and late (ORF65 and ORF75C) gene products upon a single-step infection (MOI 5). (D) Immunofluorescence of NIH 3T3 cells transfected with a FLAG-ORF75A expression construct, followed by 24 h infection with MHV68-H2BYFP (MOI of 5). (E) Quantification of ORF75A cellular localization. Two individuals independently scored at least 100 cells of each sample, for two independent sample sets. *** p ≤ 0.0005. (TIF) [file ppat.1006843.s003.tif]

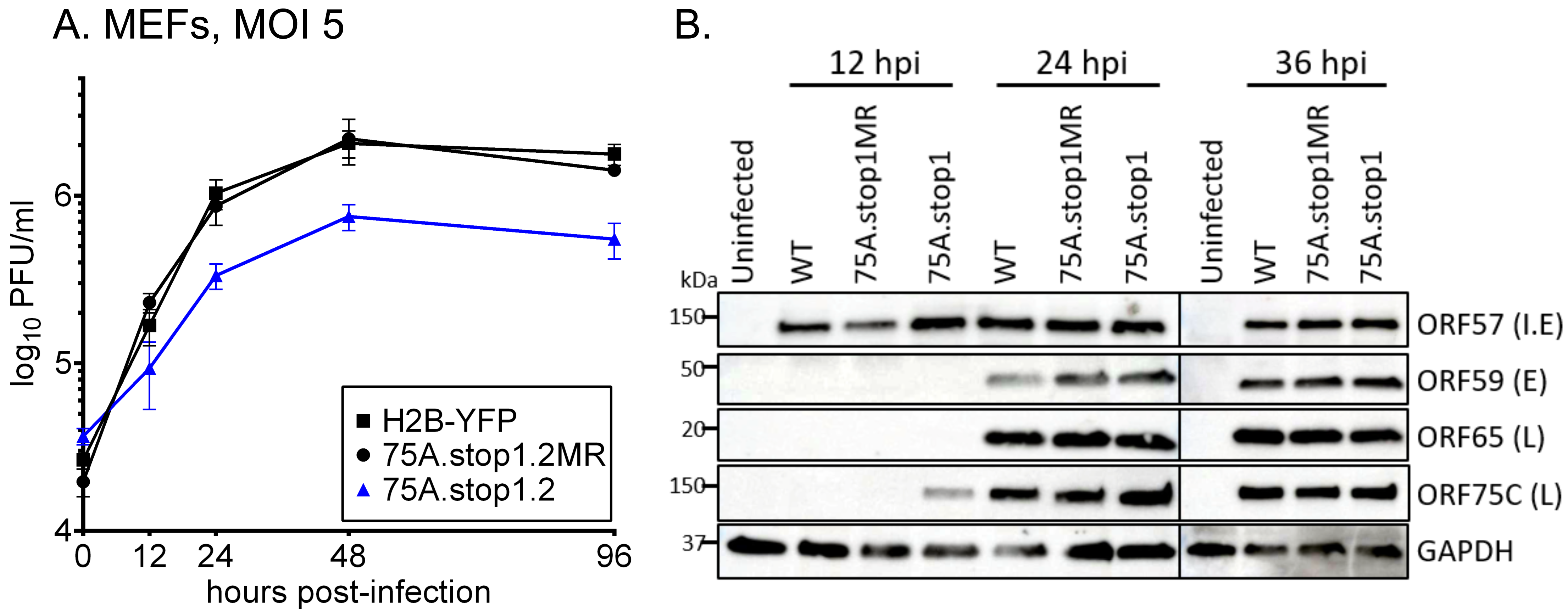

Supplement: S4 Fig — (A) Single-step growth curve in MEFs at an MOI of 5 with 75A.stop1.2 and 75A.stop1MR. (B) Timecourse analysis of gene products upon a single-step infection of MEFs. (TIF) [file ppat.1006843.s004.tif]

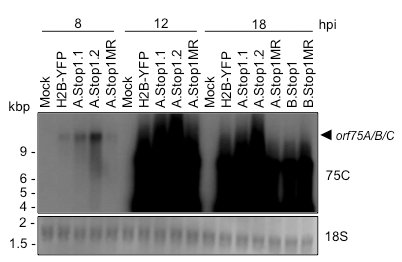

Supplement: S5 Fig — Northern blot analysis of NIH 3T12 fibroblast cells infected with indicated viruses at an MOI of 5. Membrane was hybridized with same strand-specific 32P-labled cDNA of ORF75C or 18S as described in Fig 5F. ORF75C blot was exposed for 24 hours. (TIF) [file ppat.1006843.s005.tif]

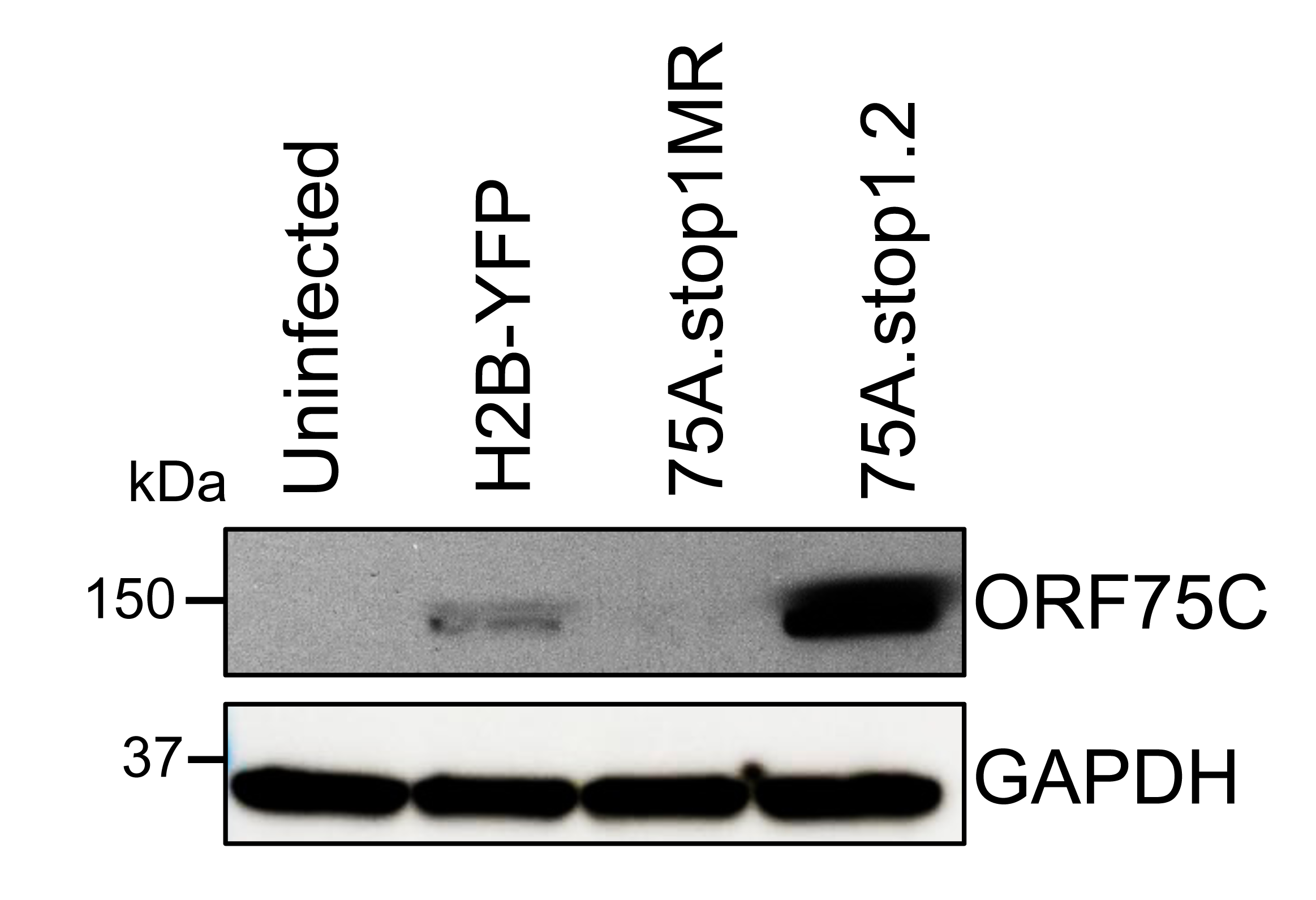

Supplement: S6 Fig — Immunoblot analysis of ORF75C tegument protein levels 3 hpi of primary BMDMs (MOI 5.0). (TIF) [file ppat.1006843.s006.tif]
