# Supplementary material for: Viral FGARAT ORF75A promotes early events in lytic infection and gammaherpesvirus pathogenesis in mice
Source: PLoS Pathog. 2018 Feb 1;14(2):e1006843. doi: 10.1371/journal.ppat.1006843 (PMC5811070; doi:10.1371/journal.ppat.1006843)
Supplement: S1 Table — (DOCX) [file ppat.1006843.s007.docx]

| **Table S1. GenBank accession numbers of vFGARATs** | | |
| --- | --- | --- |
| **GenBank Name [gammaherpesvirus]** | **Reference Name** | **Accession Number** |
| tegument protein [Alcelaphine gammaherpesvirus 1] | Alcelaphin-1 ORF3 | NP_065508.1 |
| ORF75 [Alcelaphine gammaherpesvirus 1] | Alcelaphine-1 ORF75 | NP_065571.1 |
| orf75 [Alcelaphine gammaherpesvirus 2] | Alcelaphine-2 ORF75 | YP_009044455.1 |
| orf03; similar to FGARATs [Ateline gammaherpesvirus 3] | Ateline ORF3 | NP_047978.1 |
| orf 75 [Ateline gammaherpesvirus 3] | Ateline ORF75 | NP_048047.1 |
| tegument protein/v-FGAM-synthetase [Bovine gammaherpesvirus 6] | Bovine ORF3 | YP_009041985.1 |
| FGAM-synthase [Bovine gammaherpesvirus 6] | Bovine ORF75 | YP_009042051.1 |
| ORF65 [Callitrichine gammaherpesvirus 3] | Callitrichine ORF65 | NP_733922.1 |
| tegument protein/FGARAT [Cricetid gammaherpesvirus 2] | Cricetid ORF75A | YP_004207912.1 |
| tegument protein/FGARAT [Cricetid gammaherpesvirus 2] | Cricetid ORF75B | YP_004207911.1 |
| BNRF1 [Human herpesvirus 4 type 2] | EBV BNRF1 | YP_001129438.1 |
| protein G3 [Equid gammaherpesvirus 2] | Equid-2 ORF3 | NP_042600.1 |
| ORF75 [Equid gammaherpesvirus 2] | Equid-2 ORF75 | NP_042672.1 |
| protein G3 [Equid gammaherpesvirus 5] | Equid-5 ORF3 | YP_009118394.1 |
| tegument protein G75 [Equid gammaherpesvirus 5] | Equid-5 ORF75 | YP_009118465.1 |
| ORF75 [Felis catus gammaherpesvirus 1] | Felis catus ORF75 | YP_009173956.1 |
| tegument-protein [Harp seal herpesvirus] | Harp Seal ORF75 | AJG43002.1 |
| viral phosphoribosylformylglycineamide amidotransferase [Saimiriine gammaherpesvirus 2] | HVS ORF3 | CAC84297.1 |
| viral phosphoribosylformylglycineamide amidotransferase [Saimiriine gammaherpesvirus 2] | HVS ORF75 | CAC84373.1 |
| membrane antigen p140 homolog [Human gammaherpesvirus 8] | KSHV ORF75 | AAD04748.1 |
| ORF75 [Macaca nemestrina rhadinovirus 2] | Macaca nemestrina ORF75 | AJE29733.1 |
| BNRF1 [Lymphocryptovirus Macaca/pfe-lcl-E3] | Macaca BNRF1 | ALF03207.1 |
| JM161 [Macaca fuscata rhadinovirus] | Macaca fuscata JM161 | AAT00138.1 |
| BNRF1 [Macacine gammaherpesvirus 4] | Macaine BNRF1 | YP_067940.1 |
| tegument protein/FGARAT [Murid gammaherpesvirus 4] | MHV68 ORF75A | NP_044917.1 |
| tegument protein/FGARAT [Murid gammaherpesvirus 4] | MHV68 ORF75B | NP_044916.1 |
| tegument protein/FGARATc [Murid gammaherpesvirus 4] | MHV68 ORF75C | NP_044915.1 |
| protein G3 [Myotis gammaherpesvirus 8] | Myotis ORF3 | YP_009229841.1 |
| ORF75 [Myotis gammaherpesvirus 8] | Myotis ORF75 | YP_009229907.1 |
| ORF3 [Ovine gammaherpesvirus 2] | Ovine ORF3 | YP_438131.1 |
| ORF75 [Ovine gammaherpesvirus 2] | Ovine ORF75 | YP_438197.1 |
| tegument protein/v-FGAM-synthase [Porcine lymphotropic herpesvirus 3] | Porcine ORF75 | AAO12296.1 |
| ORF75 [Retroperitoneal fibromatosis-associated herpesvirus] | RF-herpesvirus ORF75 | AGY30763.1 |
| tegument prot/FGARAT [Rhesus monkey rhadinovirus H26-95] | RRV ORF75 | AAF60074.1 |
| protein G75A [Wood mouse herpesvirus] | Wood mouse ORF75A | ACY41146.1 |
| protein G75B [Wood mouse herpesvirus] | Wood Mouse ORF75B | ACY41145.1 |
| tegument protein G75C [Wood mouse herpesvirus] | Wood Mouse ORF75C | ACY41144.1 |
