# Supplementary material for: Viral FGARAT ORF75A promotes early events in lytic infection and gammaherpesvirus pathogenesis in mice
Source: PLoS Pathog. 2018 Feb 1;14(2):e1006843. doi: 10.1371/journal.ppat.1006843 (PMC5811070; doi:10.1371/journal.ppat.1006843)
Supplement: S2 Table — (DOCX) [file ppat.1006843.s008.docx]

| **Table S2. Mutagenic primers used in the generation of the ORF75.stop viruses** | | |
| --- | --- | --- |
| **Mutant** | **Forward primer (5’ to 3’)** | **Reverse primer (5’ to 3’)** |
| 75A.stop1MR | CACCCACGAAAACCACGAATTGGGCTTTGTGGTCGTTCACACCTCGACCACTTCCTCATTTAGGGATAACAGGGTAATCGATTT | GTCTCGTGGCAGTTTCGATCAAATGAGGAAGTGGTCGAGGTGTGAACGACCACAAAGCCCAAGCCAGTGTTACAACCAATTAACC |
| 75A.stop1 | GTTCTTGCAGCGCTGTAGCTGGAGGCAAGCTTTGCAGGTGAGAGTCATGCAAAATCCAGCAATTCGGGCCTTCTGGGGAACCCCCTGGGGC | CGAGACGAATTAGGCGATGGATACAGGTGGCAGCACGCCTCACCTAGCAGCGATCTCACGCTTGATAGCACCAGAGAGCCCCCAGATGATG |
| 75A.stop2 | CACCCACGAAAACCACGAATTGGGCTTTGTGGTCGTTCACTAGTAACCTCGACCACTTCCTCATTTTAGGGATAACAGGGTAATCGATTT | GTCTCGTGGCAGTTTCGATCAAATGAGGAAGTGGTCGAGGTTACTAGTGAACGACCACAAAGCCCAGCCAGTGTTACAACCAATTAACC |
| 75A.dbl.stop | GTTCTTGCAGCGCTGTAGCTGGAGGCAAGCTTTGCAGGTGAGAGTCATGCAAAATCCAGCAATTCGGGCCTTCTGGGGAACCCCCTGGGGC | CGAGACGAATTAGGCGATGGATACAGGTGGCAGCACGCCTCACCTAGCAGCGATCTCACGCTTGATAGCACCAGAGAGCCCCCAGATGATG |
|  | CACCCACGAAAACCACGAATTGGGCTTTGTGGTCGTTCACTAGTAACCTCGACCACTTCCTCATTTTAGGGATAACAGGGTAATCGATTT | GTCTCGTGGCAGTTTCGATCAAATGAGGAAGTGGTCGAGGTTACTAGTGAACGACCACAAAGCCCAGCCAGTGTTACAACCAATTAACC |
| 75B.stop1MR | AAGAAATACTCACTGAACTHTTATCTCCCATGGGAGCATTGACATCAATGATTTGCTAGGGATAACAGGGTAATCGATTT | ACCATGACATACTGGATCTGGCAAATCATTGATGTCAATTAATGCTCCCATGGGAGATAAGCCAGTGTTACAACCAATTAACC |
| 75B.stop1 | CGAGACGAATTAGGCGATGGATACAGGTGGCAGCACGCCTCACCTAGCAGCGATCTCACGCTTGATAGCACCAGAGAGCCCCCAGATGATG | CATCATCTGGGGGCTCTCTGGTGCTATCAAGCGTGAGATCGCTGCTAGGTGAGGCGTGCTGCCACCTGTATCCATCGCCTAATTCGTCTCG |
| 75B.dbl.stop | CGAGACGAATTAGGCGATGGATACAGGTGGCAGCACGCCTCACCTAGCAGCGATCTCACGCTTGATAGCACCAGAGAGCCCCCAGATGATG | CATCATCTGGGGGCTCTCTGGTGCTATCAAGCGTGAGATCGCTGCTAGGTGAGGCGTGCTGCCACCTGTATCCATCGCCTAATTCGTCTCG |
|  | AAGAAATACTCACTGAACTGTTATCTCCCATGGGAAGCATTTTGATAATTGACATCAATGATTTGCCATAGGGATAACAGGGTAATCGATT | AGACCATGACATACTGGATCTGGCAAATCATTGATGTCAATTATCAAAATGCTCCCATGGGAGATAAGCCAGTGTTACAACCAATTAACC |

| **Table S3. Mutagenic primers used in the generation of the ORF75.stop viruses** | | |
| --- | --- | --- |
| **gBlocks** | | |
| **Mutant** | **gBlock 1** | **gBlock 2** |
| **FLAG-75A** | CGCTCCGCCGCCACACGCATTTCCTTTAACACAACACACTGAGCTAGAGCCAGGACCATGGACTACAAAGACCATGACGGTGATTATAAAGATCATGACATCGATTACAAGGATGACGATGACAAGTCAGACGACTTTATTTGGACTAGGGATAACAGGGTAATCGATTTATTCAACAAAGCCACGTTGTGTCTCAAAATCTCTGATGTTACATTGCACAAGATAAAAATATATCATCATGAACAATAAAACTGTCTGCTTACATAAACAGTAATACAAGGGGTGTTATGAGCCATATTCAACGGGAAACGTCTTGCTCGAGGCCGCGATTAAATTCCAACATGGATGCTGATTTATATGGGTATAAATGGGCTCGCGATAATGTCGGGCAATCAGGTGCGACAATCTATCGATTGTATGGGAAGCCCGATGCGCCAGAGTTGTTTCTGAAACATGGCAAAGGTAGCGTTGCCAATGATGTTACAGATGAGATGGTCAGACTAAACTGGCTGACGGAATTTATGCCTCTTCCGACCATCAAGCATTTTATCCGTACTCCTGATGATGCATGGTTACTCACCACTG | CTCCTGATGATGCATGGTTACTCACCACTGCGATCCCCGGGAAAACAGCATTCCAGGTATTAGAAGAATATCCTGATTCAGGTGAAAATATTGTTGATGCGCTGGCAGTGTTCCTGCGCCGGTTGCATTCGATTCCTGTTTGTAATTGTCCTTTTAACAGCGATCGCGTATTTCGTCTCGCTCAGGCGCAATCACGAATGAATAACGGTTTGGTTGATGCGAGTGATTTTGATGACGAGCGTAATGGCTGGCCTGTTGAACAAGTCTGGAAAGAAATGCATAAGCTTTTGCCATTCTCACCGGATTCAGTCGTCACTCATGGTGATTTCTCACTTGATAACCTTATTTTTGACGAGGGGAAATTAATAGGTTGTATTGATGTTGGACGAGTCGGAATCGCAGACCGATACCAGGATCTTGCCATCCTATGGAACTGCCTCGGTGAGTTTTCTCCTTCATTACAGAAACGGCTTTTTCAAAAATATGGTATTGATAATCCTGATATGAATAAATTGCAGTTTCATTTGATGCTCGATGAGTTTTTCTAATCAGAATTGGTTAATTGGTTGTAACACTGGCGAGCTAGAGCCAGGACCATGGACTACAAAGACCATGACGGTGATTATAAAGATCATGACATCGATTACAAGGATGACGATGACAAGTCAGACGACTTTATTTGGACATTGAGGGTCTTCCACCCATGTTCCCCGAGCGCTGAAGAT |
| **ORF75C Δ648-659** | TAGCATTATGATGTGTACTGCACTTAAAACGACCCCCCAGACCCTCCGAGTTCATAGTGATAGGGATAACAGGGTAATCGATTTATTCAACAAAGCCACGTTGTGTCTCAAAATCTCTGATGTTACATTGCACAAGATAAAAATATATCATCATGAACAATAAAACTGTCTGCTTACATAAACAGTAATACAAGGGGTGTTATGAGCCATATTCAACGGGAAACGTCTTGCTCGAGGCCGCGATTAAATTCCAACATGGATGCTGATTTATATGGGTATAAATGGGCTCGCGATAATGTCGGGCAATCAGGTGCGACAATCTATCGATTGTATGGGAAGCCCGATGCGCCAGAGTTGTTTCTGAAACATGGCAAAGGTAGCGTTGCCAATGATGTTACAGATGAGATGGTCAGACTAAACTGGCTGACGGAATTTATGCCTCTTCCGACCATCAAGCATTTTATCCGTACTCCTGATGATGCATGGTTACTCACCACTG | CTCCTGATGATGCATGGTTACTCACCACTGCGATCCCCGGGAAAACAGCATTCCAGGTATTAGAAGAATATCCTGATTCAGGTGAAAATATTGTTGATGCGCTGGCAGTGTTCCTGCGCCGGTTGCATTCGATTCCTGTTTGTAATTGTCCTTTTAACAGCGATCGCGTATTTCGTCTCGCTCAGGCGCAATCACGAATGAATAACGGTTTGGTTGATGCGAGTGATTTTGATGACGAGCGTAATGGCTGGCCTGTTGAACAAGTCTGGAAAGAAATGCATAAGCTTTTGCCATTCTCACCGGATTCAGTCGTCACTCATGGTGATTTCTCACTTGATAACCTTATTTTTGACGAGGGGAAATTAATAGGTTGTATTGATGTTGGACGAGTCGGAATCGCAGACCGATACCAGGATCTTGCCATCCTATGGAACTGCCTCGGTGAGTTTTCTCCTTCATTACAGAAACGGCTTTTTCAAAAATATGGTATTGATAATCCTGATATGAATAAATTGCAGTTTCATTTGATGCTCGATGAGTTTTTCTAATCAGAATTGGTTAATTGGTTGTAACACTGGCCACTTAAAACGACCCCCCAGACCCTCCGAGTTCATAGTGACAGTGCATTGGATCTGATC |
| **Gibbson Assembly Primers** | | |
| **Mutant** | **Forward primer (5’ to 3’)** | **Reverse primer (5’ to 3’)** |
| **FLAG-75A** | AGTGGTCTGTTCTGATGGACTGTG | TCGTGAAGTAAGGTCACTAATAGG |
| **ORF75C Δ648-659** | TAGCATTATGATGTGTACTGCACTTAAA | GTACTCAGGTGCCAGCTTGTGTAG |
