# Supplementary material for: Viral FGARAT ORF75A promotes early events in lytic infection and gammaherpesvirus pathogenesis in mice
Source: PLoS Pathog. 2018 Feb 1;14(2):e1006843. doi: 10.1371/journal.ppat.1006843 (PMC5811070; doi:10.1371/journal.ppat.1006843)
Supplement: S3 Table — (DOCX) [file ppat.1006843.s009.docx]

| **Table S3. Northern Blot Probes** | |
| --- | --- |
| **Probe** | **Primer (5’ to 3’)** |
| **ORF75A** | TCCACCCATGTTCCCCGAGCGCTGAAGATCTGGCCACCCAGAGGGGGCCGCCTCTCGCGTGGGGACAGAATCCTGGTCTGGAATGTTTCCTGCCCACGCGTGTTTTGGAGTGCAGGGCCGTACTTCCATCCTCCGAGGTGGATGACAGATCCAGAATATCTATGCTGTTCAGTAGACTGGCTGCTGCCTTCACCCGTGACCTCTCAAGACGTCACCTGGGCATGCGCCCCAGGGGGTTCCCCAGAAGGCCCGAATTGCTGGATTTTGCATACTCTCACCTGCAAAGCTTGCCTCCAGCTACAGCGCTGCAAGAACTAATCAACTCTGCAGTGGTTGGCACGCACCCTCTTCGTGCAAACATTCGCATCGACCTCACCCATCACCTATTAGTGACCTTACTTCACGAGGGATCTACGCTGGCTGAAGTTACCCACCGTGTCCTATCATACCTTCACTCTTGGAATGTTGAGGGACGGTCCCGC |
| **ORF75B** | GGCAATCCAAGTCTATGCCAGGGCCAGGACCACATTGGAGGAACATGTCCTTAACAGACTAATACTGTCAAGAACAATTCACTTTCCTCTCCGAAGCACCAGTATATGCGGTGTCCTCACCTTGACTATAAGGAACAGTCGGACCAGGAATGGGCGTGCCAGCTCGAGACGAATTAGGCGATGGATACAGGTGGCAGCACGCCTCACCTGCAGCGATCTCACGCTTGATAGCACCAGAGAGCCCCCAGATGATGGGCATGGCGATGCCCAGCGGCTAGATTTTATTTATGGACCCTTCCCTGGGCAACGGCCCCTGACCTGGTGCAATGAACTGACAGAACTCCTGGGATATATTCTTCCTATGGGAACCACGCGAATGGACTATTCTAGGAGGATATGTGGAATCTATACCCAACCAAACGCCGCCATACTTGTAATGATAGAACTACTGTACTGGTTCATATATGGCAGAGAGCGTACACAGCTGGAATATGACCGC |
| **ORF75C** | GCTAGACACTTTGCCTTTATCTATTTTGGTGATAGTCAATATAATGAGACAGAAAAAGAACTCATCGAAGACACAGAGGCTGGGAGAGCCCCCGTGGACACATCTGGTCACAGATTCATCAACATAGTTTGTGGAAGCCTTATTCCCTCCAACCCTAACAATGTTAATCATGAGCATGTTGGTATCTATAAAAGAATAATTCAACATGCGATGTCTGCTGAGTCACCACGTCTACCCGTCACTGCCACCCCGATTGATAAATCTAATAGTTCTCGAGCCCTTGCACTTTCATATGGACCTAACACCAGATGGCGACCTACCACCGTTTCCAGAGAACTGGCAGCCTATCTTCACGATCTGATTCCTGAATATAGTATCAGAATAGAATCCTTTAGGCGCGTAATCTGCACCTTGGAAAACACACCAACCAATATCTCAAACACACGACTG |
